# Potential for evolutionary responses to climate change – evidence from tree populations

**DOI:** 10.1111/gcb.12181

**Published:** 2013-04-03

**Authors:** Florian J Alberto, Sally N Aitken, Ricardo Alía, Santiago C González-Martínez, Heikki Hänninen, Antoine Kremer, François Lefèvre, Thomas Lenormand, Sam Yeaman, Ross Whetten, Outi Savolainen

**Affiliations:** * Department of Biology and Biocenter Oulu, University of OuluFIN-90014, Oulu, Finland; † UMR1202 Biodiversité Gènes et Communautés, INRAF-33610, Cestas, France; ‡ UMR1202 Biodiversité Gènes et Communautés, Université de BordeauxF-33410, Talence, France; § Department of Forest and Conservation Sciences and Centre for Forest Conservation Genetics, University of British ColumbiaVancouver, BC V6T 1Z4, Canada; ¶ Department of Forest Ecology and Genetics, INIA - Forest Research CentreE-28040, Madrid, Spain; ‖ Department of Biosciences, University of HelsinkiFIN-00014, Helsinki, Finland; ** URFM, UR629 Ecologie des Forêts Méditerranéennes, INRAF-84914, Avignon, France; †† Centre d'Ecologie Fonctionnelle et Evolutive, CNRS, Université de MontpellierUMR 5175, F-34293, Montpellier, France; ‡‡ Institute of Biology, Université de NeuchâtelCH-2000, Neuchâtel, Switzerland; §§ Department of Forestry & Environmental Resources, NC State UniversityRaleigh, NC, 27695-8008, USA

**Keywords:** adaptive traits, conifers, local adaptation, natural selection, phenotypic plasticity, provenance trials, quantitative genetics

## Abstract

Evolutionary responses are required for tree populations to be able to track climate change. Results of 250 years of common garden experiments show that most forest trees have evolved local adaptation, as evidenced by the adaptive differentiation of populations in quantitative traits, reflecting environmental conditions of population origins. On the basis of the patterns of quantitative variation for 19 adaptation-related traits studied in 59 tree species (mostly temperate and boreal species from the Northern hemisphere), we found that genetic differentiation between populations and clinal variation along environmental gradients were very common (respectively, 90% and 78% of cases). Thus, responding to climate change will likely require that the quantitative traits of populations again match their environments. We examine what kind of information is needed for evaluating the potential to respond, and what information is already available. We review the genetic models related to selection responses, and what is known currently about the genetic basis of the traits. We address special problems to be found at the range margins, and highlight the need for more modeling to understand specific issues at southern and northern margins. We need new common garden experiments for less known species. For extensively studied species, new experiments are needed outside the current ranges. Improving genomic information will allow better prediction of responses. Competitive and other interactions within species and interactions between species deserve more consideration. Despite the long generation times, the strong background in quantitative genetics and growing genomic resources make forest trees useful species for climate change research. The greatest adaptive response is expected when populations are large, have high genetic variability, selection is strong, and there is ecological opportunity for establishment of better adapted genotypes.

## Introduction

Populations can respond to environmental change through phenotypic plasticity, by moving to a new area corresponding to environmental conditions they are adapted to, by genetically adapting to the new conditions, or by combinations of these responses (Aitken *et al*., 2008). Most attention has been paid to range expansion or contraction (Parmesan, 2006; Chen *et al*., 2011), typically using models that assume the species are genetically homogenous. The potential for genetic responses has often been neglected, for instance in the IPCC reports (IPCC, 2001, 2007), even if it is well known that evolutionary changes, i.e., genetic responses, have historically accompanied changes in climate (Davis & Shaw, 2001). Furthermore, it is also now understood that the rate of adaptation required by climate change varies among geographic regions (Loarie *et al*., 2009). Modeling work on the potential of populations and species to respond genetically to recent climate change is advancing (see Hoffmann & Sgrò, 2011; Franks & Hoffmann, 2012; Shaw & Etterson, 2012 for recent reviews). The immediate responses via phenotypic plasticity have also been considered in the context of climate change (Nicotra *et al*., 2010).

Here, we examine the importance of and potential for genetic responses to climate change in forest tree populations. Trees are ecologically key species in many terrestrial ecosystems, including boreal and temperate forests in Europe and North America. Their response to climate change can substantively impact the global carbon cycle. Local adaptation (Kawecki & Ebert, 2004) is more common in trees than in some other plant species. Tree species are adapted to the current climate, and they are thus potentially greatly influenced by the rapid changes in climate (Savolainen *et al*., 2007). The long generation times are a challenge for research, but trees also provide some advantages for these studies, as described below.

First, adaptation to climate change will depend on phenotypic traits relevant in the new environments, such as timing of growth and drought or cold tolerance. There is an extraordinary wealth of information on the quantitative genetics and population differentiation of trees for these traits, based on 250 years of forestry common garden experiments, known as provenance trials (Langlet, 1971; Morgenstern, 1996), and on extensive tree breeding experience.

Second, the demographic history since the last glacial maximum has been reconstructed for several tree species by combining phylogeographic and palynological approaches with coalescent-based studies of population demography (Petit *et al*., 2002; McLachlan *et al*., 2005; Cheddadi *et al*., 2006; Heuertz *et al*., 2006; Magri *et al*., 2006; Soltis *et al*., 2006; Eckert *et al*., 2010; Parducci *et al*., 2012). Rates of past adaptation of trees to climate changes can be inferred from these studies (Hendry & Kinnison, 1999). The increasing knowledge of the molecular basis of quantitative trait variation (see Neale & Kremer, 2011 for references) can improve predictive models (see e.g., Wilczek *et al*., 2010). This body of background information allows us to examine the potential for adaptation in natural conditions better than in many other organisms. For instance, in butterflies, studies of responses to climate change have relied nearly exclusively on examining molecular marker variation (Hill *et al*., 2011).

Trees have very long generation times, but they share population genetic characteristics with other outcrossing plants and animals with high levels of gene flow and large effective population sizes (Petit & Hampe, 2006). Trees are highly fecund, and may rapidly increase their population sizes. Because they are sessile, they generally have good tolerance of a range of environmental conditions and large plastic responses. There are ecologically and commercially important trees with large continuous distributions, such as *Picea abies*, *Pinus contorta*, and *P. sylvestris*, but also species with small, fragmented distributions more susceptible to genetic drift. The dispersal capacity of tree species will play a crucial role in their potential for adaptation. Hybridization between closely related tree species can also influence their adaptive capacity out of their current range, as it has been shown in other organisms (Hoffmann & Sgrò, 2011; Olson-Manning *et al*., 2012 and references therein).

The focus of this review was on predicting evolutionary responses, with as much evolutionary, genetic, and ecological realism as possible. We examine the models needed for prediction, starting with the simplest models of evolution in individual populations, and continuing to more complex and more realistic models involving multiple populations in heterogeneous environments. We discuss what data are needed for realistic prediction of genetic responses, what information is already available, and what additional information we need in terms of new models, new data, or new analyses of existing data (Lindner *et al*., 2010). Quantitative genetic models of evolutionary response deal with traits that will confer adaptation to future environments. While it is not easy to predict what traits will be most important in the future, it is reasonable to examine traits related to climate, such as the timing of growth and reproduction (Rohde & Bhalerao, 2007; Hänninen & Tanino, 2011) or cold and drought tolerance (Niinemets, 2010).

## Evolution in one isolated population

### A single population: the breeder's equation

**Fig. 1:**
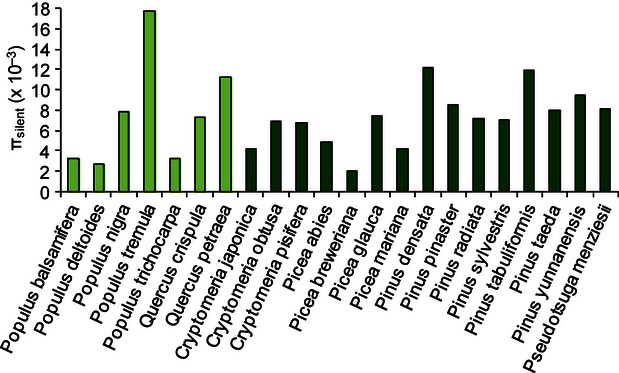
Mean silent nucleotide diversity per site (π_silent_) estimates for several tree species. Average nucleotide diversity at silent sites (for more details and references see Table S2). Angiosperms appear in light color and conifers in dark color.

According to the breeder's equation, the simplest model governing response to directional selection on a single trait, the response in a large population with no gene flow depends on the strength of selection, on the amount of genetic variation, and its ratio to total phenotypic variation (heritability; see Falconer & Mackay, 1996). If there is no genetic variation, any change in phenotype in a novel environment inducing directional selection would be due to phenotypic plasticity alone. Forest tree populations harbor considerable genetic variability in many quantitative traits (Cornelius, 1994; Morgenstern, 1996; Howe *et al*., 2003) as well as at the DNA level (see Fig. 1 and Savolainen & Pyhäjärvi, 2007). While tree breeders can control the intensity of selection and predict responses in breeding populations, it is much more difficult to make such predictions in the wild. Environmental variances will be higher, and heritabilities generally lower (Conner *et al*., 2003). Methods for estimating heritabilities in the wild are improving because of much better estimates of relatedness (Ritland, 1996; Sillanpää, 2011), and will be of critical importance to understanding responses to climate change.

Assessing the strength of directional selection is a demanding task, as we do not even know exactly which traits are most important for fitness, and the longevity of trees makes lifetime fitness estimates unattainable in a realistic timeframe. Estimates of directional selection are available for natural populations (Kingsolver *et al*., 2001; Kingsolver & Diamond, 2011), but studies on forest trees are lacking. Furthermore, selection is likely to be variable across environments, years, and life stages. In natural populations, the traits are also subject not just to directional selection but also to stabilizing and disruptive selection, not included in this simplest model. Thus, for most natural situations, the breeder's equation is far from the reality of populations responding to natural selection.

### Temporal variation in selection

Two general classes of quantitative genetic models have been developed to study the risk of extinction in single populations: models with a sudden single step-change in the optimum phenotype (Pease *et al*., 1989; Gomulkiewicz & Holt, 1995; Gomulkiewicz & Houle, 2009; Gomulkiewicz *et al*., 2010), and models with a continuous change in the optimum phenotype (Lynch & Lande, 1993; Burger & Lynch, 1995; Björklund *et al*., 2009; Chevin *et al*., 2010). In single step-change models, extinction occurs as a consequence of decreasing population size due to selective deaths as the population adapts to the change in environment. In the continuous-change models, by contrast, extinction is assumed to occur when the pace of adaptation lags behind the rate of change in the optimum phenotype (see Aitken *et al*., 2008 for further discussion). There are several interesting ways in which these models could be extended to increase biological realism. Most of these models assume that the strength of selection does not vary with population density, which is unrealistic for most forest trees, as competition is likely greatly reduced at low densities (see Björklund *et al*., 2009 for a simulation model incorporating density dependent selection). Also, failing to account for changes in biotic interactions that may be associated with climatic change could cause models to under- or overestimate extinction risks (Gilman *et al*., 2010). Climate change may result in the introduction of new pests, as for instance the mountain pine beetle (Robertson *et al*., 2009) or new pathogens (Netherer & Schopf, 2010), but also losses of current competitors, insects, or diseases caused for example by phenological shifts between trees and associated pests (van Asch & Visser, 2007).

While it is possible to parameterize some of these models to make quantitative predictions about extinction risk, the assumptions involved greatly limit the faith that should be placed in any such predictions (see Aitken *et al*., 2008 for further discussion). Rather, they seem most useful as heuristic tools to identify the most likely factors causing population extinction and to compare relative risk among species. In general, these models find that the probability of extinction decreases for species with large population sizes, high fecundity, high heritabilities, and high amounts of standing genetic variation. While many forest trees present such characteristics, extra effort should be made to study species that are on the low end of the spectrum for any of these characteristics. Some examples of species that may be particularly vulnerable due to their small population sizes are *P. torreyana* in North America, or *A. pinsapo* in Europe. More study is necessary to see whether such vulnerable species also have lower levels of standing variation.

### Genetic basis of adaptive trait variation

**Fig. 2:**
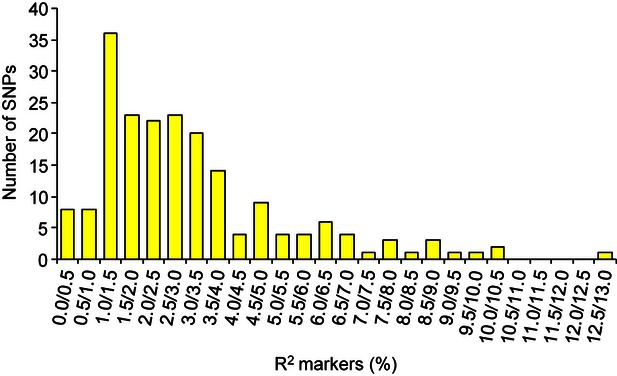
Distribution of allelic effect sizes in tree species. Distribution of the percentages of phenotypic variance explained by genotypic classes at SNP loci (*R*² marker) detected in significant associations with quantitative traits (for more details and references see Table S3).

The expected genetic responses in many models depend on the genetic architecture of the trait (e.g., Gomulkiewicz *et al*., 2010). While the traditional polygenic model of Fisher (Fisher, 1918, 1930) is based on small effects at a very large number of loci, some models of selection predict larger effect sizes (Orr, 1998; Yeaman & Whitlock, 2011). Overall, quantitative trait locus (QTL) studies in forest trees have generally found large numbers of loci with relatively small effect sizes, compared with some crop plants (Barton & Keightley, 2002; Howe *et al*., 2003; Laurie *et al*., 2004). Association studies have further confirmed this view of moderate effect sizes (summarized in Fig. 2), e.g., in *P. taeda* (Quesada *et al*., 2010; Cumbie *et al*., 2011), *Populus tremula* (Ingvarsson *et al*., 2008), *P. sitchensis* (Holliday *et al*., 2010a), and *Pseudotsuga menziesii* (Eckert *et al*., 2009). These findings are consistent with the small effect sizes of flowering time and leaf trait variation loci in maize (Buckler *et al*., 2009; Tian *et al*., 2011), and human height (Hill *et al*., 2008). In contrast, Atwell *et al*. (2010) found large effect SNPs for many phenotypic traits of *Arabidopsis*. There may also be major effect loci for disease resistance, such as for rust disease caused by fungal pathogens in North American conifers (Kayihan *et al*., 2010). The associated loci may well differ between environments due to genotype by environment interactions (Jermstad *et al*., 2003) or because of different genetic basis in different areas (Goldstein & Holsinger, 1992; Hancock *et al*., 2011). In many conditions, the phenotypic differences between populations can be due to combined effects of several loci rather than differentiation at the level of individual loci (Latta, 1998; LeCorre & Kremer, 2003; Kremer & Le Corre, 2012).

Weak genetic correlations allow traits to respond to selection independently, whereas genetic correlations opposing the direction of selection will delay the response (Etterson & Shaw, 2001), and reinforcing correlations will accelerate it. Under stabilizing selection, responses are facilitated, if the selection is weak (Duputie *et al*., 2012). The underlying causes of genetic correlations are so far not known in trees.

Overall, the limited findings so far suggest that the response to strong selection on phenotypes will often be based on many loci with small effects, and fairly weak selection on individual loci, as has been also found in humans (Turchin *et al*., 2012). If larger effect loci are involved, response predictions could then use specific information on such loci. Alternatively, genomic selection methods could be used to build predictive models that do not need to identify the particular loci underlying adaptive genetic responses (Grattapaglia & Resende, 2011; Iwata *et al*., 2011; Holliday *et al*., 2012; Resende *et al*., 2012).

We do not know whether most adaptations in trees are due to existing variation or new mutations. During interglacial periods, tree populations have repeatedly colonized northern areas and have rapidly adapted to those conditions, likely because the north-adapted variants may have remained in southern populations at lower frequencies (De Carvalho *et al*., 2010; Savolainen *et al*., 2011). Typically, large effective population sizes in forest trees would have contributed to rapid fixation of adaptive variants. This supports an interpretation of evolution from standing rather than *de novo* variation.

### Phenotypic plasticity and adaptation

Trees exhibit a high degree of phenotypic plasticity with respect to climatic variation. Phenological shifts of bud flush in response to recent increases in temperatures have been widely documented (Menzel & Fabian, 1999; Menzel *et al*., 2006; Parmesan, 2006). Arid years or an arid microsite may favor the development of deeper and denser root systems (Kozlowski & Pallardy, 2002). In such a context, adaptive plasticity can buffer the impact of changing conditions on population size (Lynch & Lande, 1993). However, these plastic changes may take time to develop, as in the root example above. In addition, more plasticity also means less intense selection, causing populations to genetically track changing optima more slowly. Recent models have shown that the decreased selection is more than compensated for by the increased phenotypic match allowed by plasticity (Chevin & Lande, 2010). In fact, the evolution of plasticity can provide populations with a transient and efficient response to large environmental changes (Lande, 2009).

Multiple-site provenance trials can be used to examine the plastic responses of populations in new environments. This can be quantified with response functions for individual populations, which describe the change in a trait as a function of transfer distance or change in environmental factors (Rehfeldt *et al*., 1999, 2002). Provenance trials have been planted in sites that vary with respect to many environmental variables, such as temperature or water availability (Morgenstern, 1978; Kramer, 1995; Shutyaev & Giertych, 1997; Rehfeldt *et al*., 1999, 2002; Worrell *et al*., 2000; Reich & Oleksyn, 2008; Vitasse *et al*., 2010). Transfers to the south have been used to predict responses to a warming climate (Beuker *et al*., 1998; Rehfeldt *et al*., 2002; Wang *et al*., 2006) even if the future conditions may be different (e.g., photoperiod). Furthermore, these experiments take place in spaced plantings of seedlings, and thus ignore germination, establishment, and early intra- and interspecific competitive effects. Response functions of individual populations have been developed for growth using very large datasets of multiple trials including more than a hundred populations available for *P. contorta* (Rehfeldt *et al*., 2001), *P. sylvestris* (Rehfeldt *et al*., 2002), and *Larix occidentalis* (Rehfeldt & Jaquish, 2010). Recently, Wang *et al*. (2010) developed a universal response function for *P. contorta*, which integrated populations and environment effects and can be used to predict the performance of any population in any climatic conditions. Incorporating provenance trial data on local adaptation and phenotypic plasticity in models predicting future distributions reduced dramatically the extinction risk in southern populations (Morin & Thuiller, 2009; Benito-Garzón *et al*., 2011). The plastic response of different traits (e.g., phenology in trees) to variation in climate is, however, often much more complex than in heuristic models of adaptation (see e.g., Valladares *et al*., 2007; Caffarra *et al*., 2011; Hänninen & Tanino, 2011).

Finally, epigenetic effects on phenotypic plasticity and inheritance of phenotypic variation need further investigation. Epigenetic variation can be partly inherited from one generation to the next while being still sensitive to environmental variation (Richards *et al*., 2010). Maternal epigenetic effects are known in *Arabidopsis* (Johannes *et al*., 2009), but so far their nature has not been studied much in trees (Bräutigam *et al*., 2013). Epigenetic effects can also occur during seed maturation. Temperature differences during embryogenesis caused differences in phenology in *P. abies* (Skrøppa & Kohmann, 1997) and the molecular mechanisms involved are being studied (Yakovlev *et al*., 2010). They could have significant implications for the interpretation of provenance trial data, explaining some of the phenotypic variation among populations that is commonly interpreted as genetic variation.

## Evolution in multiple populations

### Geographic distribution and genetic structure

**Fig. 3:**
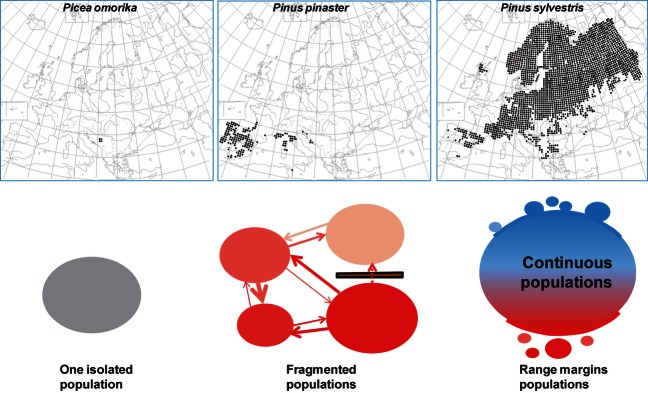
Schemes of the population models used to discuss evolutionary responses. The three different schematic models of population structure encountered in tree species illustrated by the different cases of *Picea omorika* (one limited population), *P. pinaster* (several fragmented populations) and *P. sylvestris* (large and continuous population). The color of the circle indicates the environmental condition of the population which is either undefined (in gray) or following a temperature gradient from warm (in red) to cold (in blue). The arrows represent gene flow connecting populations, with thickness indicating gene flow intensity. For the fragmented populations, the brown line symbolizes a physical barrier to gene flow, such as a mountain.

Natural populations of a species in a heterogeneous landscape may have very different patterns of distribution, which can influence its population genetic characteristics (Fig. 3) as reviewed by Charlesworth & Charlesworth (2010). The classical island model assumes populations of equal finite constant size, with equal migration rates between them (Wright, 1931). These assumptions can be relaxed, with variable migration rates and changing population sizes. Species can also be distributed in large continuous populations where parts of the range are connected by symmetric gene flow, as described in the isolation by distance model by Wright (1943). Populations located at range margins represent a special case, as they are at the edge of environmental gradients where carrying capacity may be limited. In such cases, there is more migration from the core populations to the margin than *vice versa*, resulting in asymmetric gene flow (Kirkpatrick & Barton, 1997).

Many economically important temperate and boreal species have large populations covering vast areas, but other tree species do not fit this distribution model. We examined the population structure of European conifers in the *Pinaceae* (including pines, spruces and firs), a limited group of species with very good distributional and reasonable population genetics information. A compilation of the distributions of these 27 species (and sometimes subspecies; from Jalas & Suominen, 1973), allowed us to classify them as having northern or central large, southern large or southern small or fragmented distributions (Table 1). Note that the classification is based on the current distributions, although some currently fragmented species may have had much larger distributions in the past (Soto *et al*., 2010). Species with a predominantly northern distribution, but also occurring in the south (e.g., *P. sylvestris*) were classified as northern species. Figure 3 shows examples of distributions of three species (*P. omorika, P. pinaster*, and *P. sylvestris*). There are 11 species with predominantly northern or central, large, continuous distributions, and four southern species with large, but somewhat fragmented distributions. About half of the European conifers (12) have southern, small, or fragmented distributions. Furthermore, the southern margin of most species seems to consist of fragmented small populations, whereas in the north, the range margin is part of a continuous distribution for several species. This analysis shows that in many tree populations, the threats associated with climate change are accompanied by and likely exacerbated by the effects of fragmentation at southern range margins (see also Lynch, 1996). However, if there is still extensive gene flow among the fragments, the population structure should resemble that of a continuous population.

**Table 1:** Distribution range and genetic estimates for the 27 European conifers

Species	Range	Distribution	Mean *Q*_ST_*	*Q*_ST_ range*	*F*_ST_	*H*_e_	Reference †
*Abies nebrodensis*	Sicilia	South small				0.201	Ducci *et al*. (1999)
*Abies pinsapo*	Andalusia	South small				0.056	Scaltsoyiannes *et al*. (1999)
*Pinus nigra ssp dalmatica*	South Croatia	South small			0.091	0.292	Nikolic & Tucic (1983)
*Picea omorika*	Croatia Serbia	South small			0.261	0.067	Ballian *et al*. (2006)
*Pinus nigra ssp laricio*	Corsica Calabria Sicilia	South small			0.005	0.182	Scaltsoyiannes *et al*. (1994)
*Abies cephalonica*	Balkans	South small	0.140	0.100–0.170	0.048	0.221	Fady & Conkle (1993)
*Pinus peuce*	Balkans	South small			0.083	0.124	Zhelev & Tsarska (2009)
*Pinus brutia*	Aegean Sea	South fragmented	0.040		0.053	0.196	Kara *et al*. (1997)
*Pinus heldreichii*	Balkans	South fragmented			0.054	0.177	Boscherini *et al*. (1994)
*Abies borisii-regis*	Balkans	South fragmented				0.273	Scaltsoyiannes *et al*. (1999)
*Pinus nigra ssp pallasiana*	Greece Serbia Bulgaria	South fragmented	0.028	0.020 - 0.040	0.070	0.114	Tolun *et al*. (1999)
*Pinus nigra ssp salzmannii*	East Spain South France	South fragmented				0.216	Scaltsoyiannes *et al*. (2009)
*Pinus nigra ssp nigra*	North Italy Croatia Greece	South large fragmented				0.264	Scaltsoyiannes *et al*. (2009)
*Pinus pinaster*	South West Europe	South large fragmented	0.616	0.441–0.791	0.076	0.142	Salvador *et al*. (2000)
*Pinus pinea*	South Europe	South large fragmented			0.279	0.011	Fallour *et al*. (1997)
*Pinus halepensis*	South Europe	South large fragmented	0.130			0.040	Schiller *et al*. (1986)
*16 species with small or fragmented range*			0.192		0.082‡	0.171‡	
*Pinus cembra*	Alps Romania	North large continuous	0.830		0.040	0.081	Belokon *et al*. (2005)
*Pinus uncinata*	Central West Europe	North large continuous			0.006	0.260	Lewandoski *et al*. (2000)
*Larix decidua*	Central Europe	North large continuous			0.051	0.223	Maier (1992)
*Pinus sibirica*	East Siberia	North large continuous			0.027	0.278	Goncharenko *et al*. (1992)
*Pinus mugo*	Central East Europe	North large continuous			0.041	0.214	Slavov and Zhelev (2004)
*Abies alba*	Central Europe	North large continuous	0.075	0.000–0.150		0.252	Ducci *et al*. (1999)
*Abies sibirica*	Siberia	North very large continuous			0.102	0.083	Semerikova & Semerikov (2006)
*Larix sibirica*	Siberia	North very large continuous			0.082	0.159	Semerikov *et al*. (1999)
*Pinus abies ssp obovata*	Lapland Siberia	North very large continuous			0.011	0.213	Krutovskii & Bergmann (1995)
*Pinus abies ssp abies*	North Central Europe	North very large continuous	0.417	0.106 - 0.727	0.044	0.252	Krutovskii & Bergmann (1995)
*Pinus sylvestris*	Whole Europe	North very large continuous	0.519	0.080 - 0.860	0.033	0.286	Goncharenko *et al*. (1994)
*11 species with continuous range*			0.463		0.044	0.209	

* Mean *Q*_ST_ and *Q*_ST_ range were calculated from estimates only for height increment, bud flush, and bud set (for more details and references see Table S1). Q_ST_ estimates corresponds to the levels of population differentiation measured either as the proportion of phenotypic variation between populations (*V*_pop_) or as the proportion of additive genetic variance between populations (*Q*_ST_) in the provenance trials (for more details see Table S1).

† References of the studies using allozyme markers to assess *F*_ST_ and *H*_e_. See supporting information references for full reference information.

‡ *Pinus pinea*, which has hardly any within-population variation (Vendramin *et al*., 2008), was not included in the calculation of mean *F*_ST_ and mean *H*_e_.

Consistent with the theoretical predictions, the European conifers with continuous distributions have higher genetic diversity (*H*_e_) than the fragmented ones (Table 1). The widespread northern species such as *P. abies* and *P. sylvestris* have low levels of genetic differentiation (*F*_ST_) in their main range (Heuertz *et al*., 2006; Pyhäjärvi *et al*., 2007). Similar findings have been made in North America for species such as *P. menziesii* (Eckert *et al*., 2010), *P. sitchensis*, *P. glauca*, and *P. mariana* (Namroud *et al*., 2008; Chen *et al*., 2009; Holliday *et al*., 2010a,b). In contrast, the level of population differentiation is almost twice for the southern fragmented species compared with the northern widely distributed ones (Table 1). Thus, the genetic data available are broadly consistent with the population structure classification based on species distribution and census size. However, current census size may ignore effects of past demographic history such as population size changes or hybridization, and we do not expect the current distributions to account for all variation in patterns of diversity.

Next, we examine the patterns of quantitative genetic variation in tree species in general and in these European conifers in particular to evaluate the effects of selection for local adaptation. We reviewed the literature of provenance trials and found a total of 112 studies on 19 relevant traits related mostly to phenology, growth, cold or drought tolerance or other ecophysiological traits, among which were 36 studies on European conifers (Table S1). Among 59 tree species studied, most were native to Europe and North America (23 and 29 species, respectively) while conifers were more studied than angiosperms (36 and 23 species, respectively). Only three traits had been measured in a sufficiently large number of experiments (Table 2) to make general comparisons and draw general patterns. We focused on the patterns of genetic variation for height increment and for the timing of bud flush, at the beginning of the growing season in spring, and the timing of bud set, an indication of cessation of growth in fall. Among all studies, these three traits had comparable levels of genetic differentiation between populations (mean value equal to 0.249, 0.324, and 0.392 for bud flush, height increment, and bud set, respectively; Table 2).

**Table 2:** Genetic differentiation (*Q*_ST_) estimates for the 19 quantitative traits studied in provenance trials

		Q_ST_ estimates*			Qualitative estimation †	
			
Trait	Category	Mean Q_ST_	Q_ST_ range	Nb‡	Trend	Nb‡
Dark respiration	Ecophysiology			0	Moderate	2
Leaf mass per area	Ecophysiology	0.022	0.000 – 0.044	2	Variable	6
Net assimilation	Ecophysiology	0.045	0.015 – 0.075	2	Variable	8
Nitrogen leaf content	Ecophysiology	0.042	0.000 – 0.083	2	Variable	6
Photosynthetic capacity	Ecophysiology	0.101	0.000 – 0.201	2	Variable	1
Stomatal conductance	Ecophysiology	0.061	0.000 – 0.150	4	Variable	4
Stomatal density	Ecophysiology	0.028	0.000 – 0.056	2	Low	5
Water use efficiency (A/gs)	Ecophysiology	0.075		1	Variable	7
Water use efficiency (δ^13^C)	Ecophysiology			0	Variable	6
Fall frost hardiness	Frost hardiness	0.581	0.030 – 0.890	9	High	10
Spring frost hardiness	Frost hardiness	0.126	0.000 – 0.352	4	Variable	3
Winter frost hardiness	Frost hardiness	0.170	0.000 – 0.291	3		0
Growth rate per day	Growth	0.284	0.050 – 0.710	8	Moderate	3
Height increment	Growth	0.324	0.040 – 0.880	36	High	33
Root allocation	Growth	0.340	0.251 – 0.430	2	Moderate	4
Bud flush	Phenology	0.249	0.000 – 0.700	24	Moderate	37
Bud set	Phenology	0.392	0.040 – 0.904	16	High	16
Germination	Phenology	0.521	0.200 – 0.940	6	High	3
Senescence	Phenology	0.108	0.080 – 0.180	5	Low	3

* *Q*_ST_ estimates corresponds to the levels of population differentiation measured either as the proportion of phenotypic variation between populations (*V*_pop_) or as the proportion of additive genetic variance between populations (*Q*_ST_) in the provenance trials (for more details see Table S1).

† Qualitative estimation of genetic differentiation between populations corresponds to studies where no Q_ST_ estimate was available, but significance of genetic differentiation was mentioned in the text.

‡ Nb, number of studies used to calculate mean Q_ST_ and Q_ST_ range, and the trend of population differentiation.

### Quantitative variation in fragmented populations

In Europe, small and fragmented conifer populations occur mainly in the southern Mediterranean area. Provided population sizes are sufficiently large, species with greater differences among populations in local phenotypic optimum and higher levels of genetic variance would be expected to have higher equilibrium differentiation. Gene flow in contrast, would reduce differentiation (Hendry *et al*., 2001). In general, if there is strong differential selection between populations, we would expect that the proportion of total genetic variance found between populations, *Q*_ST_, should be higher than *F*_ST_ calculated from neutral markers with appropriate mutation rates (Leinonen *et al*., 2008; Edelaar *et al*., 2011).

In the limited set of provenance trials on European conifers, estimates of quantitative genetic differentiation among populations for species with small or fragmented range were low over all traits (mean *Q*_ST_ = 0.192, five species; Table 1). This average is about twice as high as the neutral *F*_ST_ (0.082; nine species; Table 1). Even though sampling across an environmental gradient is clearly not concordant with the assumptions of the island model, data of this kind are frequently analyzed by comparing *Q*_ST_ and *F*_ST_ estimates for distinct samples from large and continuous populations. The average *Q*_ST_ estimate for large populations in northern areas is 0.463 while average *F*_ST_ is 0.044. Thus, in this small set of studies, the ratio of *Q*_ST_ to *F*_ST_ is much lower for species with small or fragmented range than that found in more widespread species. In small populations or fragments, selection for local adaptation is less efficient because of the effects of genetic drift on individual loci, and further, on the associations of alleles at different loci (Le Corre & Kremer, 2012). A review by Leimu & Fischer (2008) found that in plants only about 50% of all population pairs in reciprocal transplantations studies showed evidence of local adaptation, i.e., each population at its native site had higher fitness than other populations introduced to that site. Local adaptation was much less likely in small than large populations. However, *Q*_ST_ values could also differ because the studies on species with limited distributions have sampled a smaller range of environmental variation than studies in species with large ranges, or because the scale of fragmentation does not match the scale of environmental variation. Reciprocal transplant experiments are needed to assess the level of local adaptation directly. In the large provenance trial data set over all 19 traits and 59 tree species, 264 of 294 analyses (around 90%) showed significant differentiation across populations (Table S1), in most cases likely due to climatic selection.

There is also some evidence in the literature for local climatic adaptation in southern European fragmented populations, such as for water use efficiency in *P. halepensis* (Voltas *et al*., 2008). Furthermore, some allelic variants at candidate loci for drought tolerance have also been found to be associated with environmental variables (Grivet *et al*., 2011). In some of these species, selection may have been strong enough for local adaptation to evolve. Clearly, more studies on the patterns of local adaptation are needed in the species with fragmented southern distributions. Forests at Mediterranean southern limits are threatened by faster changes in precipitation than in the northern range limit. If indeed their adaptive capacity is lower, this could make southern fragmented populations even more vulnerable.

It is also possible that these populations have evolved high adaptive phenotypic plasticity in response to environmental variability instead of genetic differentiation, either for some specific traits or across the genome (Nicotra *et al*., 2010). This could be likely if there is also a strong temporal component of environmental variation (Hedrick, 2006). In a changing climate, the responses due to phenotypic plasticity may maintain fitness despite climatic changes. More growth chamber or reciprocal transplant experiments will be needed to assess the response functions for these species.

### Quantitative variation in continuous populations along environmental gradients

**Fig. 4:**
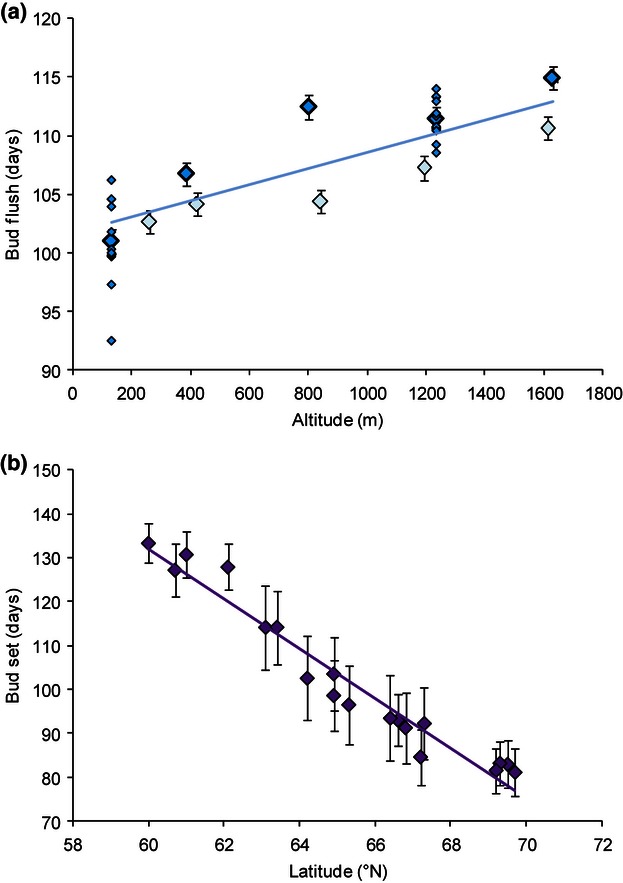
Clines of phenological traits along environmental gradients. (a) Timing of bud flush along an altitudinal gradient in *Quercus petraea*, based on data from Alberto *et al*. (2011). The timing of bud flush is expressed as the number of days from 1st January to reach the fourth developmental stage of leaf unfolding. Means of populations (large diamonds) are plotted against the altitude of origin. Bars represent standard deviations of the populations. Means of maternal tree progenies (small diamonds) in populations located at 131 m and 1235 m of elevation illustrate high additive genetic variance within populations, slightly decreasing with increasing altitude. Dark colored points represent populations and maternal trees from Luz valley while light colored points represent populations from Ossau valley. (b) Timing of bud set along a latitudinal gradient in *P. sylvestris*, based on data from Mikola (1982). The timing of bud set is measured as the number of days from the day of sowing. Means of populations (large diamonds) are plotted against latitude of origin. Bars represent standard deviations of the populations.

Species present in Central and Northern Europe generally have continuous distributions covering large areas encompassing much heterogeneity in abiotic and biotic environmental factors with large effective population sizes connected by extensive gene flow. If there is differential selection along environmental gradients, we expect to see patterns of clinal variation in traits (Barton, 1999). These patterns can be described by the slope of a regression along an environmental gradient. The proxies for environmental gradients most frequently used are latitude and altitude. For height increment, populations from warmer environments generally grew faster in the provenance trials (see Table S1), but quantitative estimates of the slopes were rarely available. Populations from cold environments cease growth earlier, as an adaptation to the approaching winter (see e.g., Savolainen *et al*., 2004). To compare slopes of clinal variation, we focused on the two phenological traits, the timing of bud flush and the timing of bud set, and compared altitudinal and latitudinal clines. To summarize data across species and environments, we considered that one degree of latitude corresponds approximately to a temperature change of 0.6 °C, and correspondingly, 100 m of altitude (Jump *et al*., 2009). We show examples of an altitudinal cline in bud flush in *Q. petraea* (Fig. 4a) and a latitudinal cline in bud set in *P. sylvestris* (Fig. 4b).

The results of the summary in Table 3 show that the two phenological traits differ in their patterns. For bud flush, both altitudinal and latitudinal clines showed similar shallow slopes, but the direction of adaptation varied greatly among species (Table 3a). For example, high altitude populations from the same transect flushed late in *Q. petraea* (Fig. 4a), whereas in *F. sylvatica* they flushed early (Vitasse *et al*., 2009). This could reflect different compromises in the adaptive tradeoff between maximizing the growing season length and exposing new leaves to late frosts. Bud flush is triggered by the accumulation of cold (or chilling) sums followed by heat (or forcing) sums above a threshold temperature sum. These genetically determined critical temperature sums and thresholds may vary among species, and to a lesser extent among populations of the same species (Hänninen & Tanino, 2011). Bud flush in late successional species is also more influenced by photoperiod than in early successional species (Körner & Basler, 2010; Basler & Körner, 2012). Bud set showed steeper slopes for both gradients and in all species more northern or higher altitude populations had earlier bud set (Table 3b). These data suggest that differential selection on bud set is systematically stronger than on bud flush. Bud flush may display higher phenotypic plasticity as temperatures increase. In contrast, bud set is largely governed by photoperiods, and modulated by temperatures and drought, which results in a more predictable environmental signal from year to year and location to location (Böhlenius *et al*., 2006). In a warming climate, spring phenology can likely respond and advance without much genetic change, as has already been seen in many species (Gienapp *et al*., 2008), provided that the chilling requirement has been met. However, if chilling temperature requirements have not been met, in some cases bud flush may even be delayed (Hänninen & Tanino, 2011), as already seen recently in Tibet (Yu *et al*., 2010). In the fall, a change in bud set date is more likely to require a genetic change in photoperiodic responses. Some studies suggest that the heritability of bud flush is higher than for bud set (Howe *et al*., 2003), but estimates of the additive genetic component are rarely available in the literature. The latitudinal slopes were also much steeper than the altitudinal ones (Table 3b). Sundblad & Andersson (1995) have suggested that along the altitudinal gradients there may be more gene flow so populations do not become as differentiated. The simple calibration factors we used also may not capture all aspects of the environment.

**Table 3:** Slopes of the linear regressions of (a) bud flush and (b) bud set along altitudinal, and latitudinal gradients

Gradient	Species	Pop *	Cline	Slope	Reference
(a)					
Altitudinal	*Abies amabilis*	5	High early	−1.18	Worrall (1983)
	*A. lasiocarpa*	2	High early	−0.83	Worrall (1983)
	*Fagus sylvatica*	9	High early	−0.43	Vitasse *et al*. (2009)
	*F. sylvatica*	158	High early	−0.17	Von Wuehlisch *et al*. (1995)
	*Pseudotsuga menziesii*	7	High early	−4.38	Acevedo-Rodriguez *et al*. (2006)
	*P. menziesii*	18	No cline	0.00	Rehfeldt (1978)
	*Picea abies*	23	No cline	−0.22	Chmura (2006)
	*P. abies*	8	No cline	−0.03	Skroppa & Magnussen (1993)
	*A. alba*	6	No cline	−0.20	Vitasse *et al*. (2009)
	*Acer pseudoplatanus*	7	No cline	−0.20	Vitasse *et al*. (2009)
	*Fraxinus excelsior*	9	Low early	1.90	Vitasse *et al*. (2009)
	*Larix occidentalis*	82	Low early	0.23	Rehfeldt (1982)
	*Quercus petraea*	10	Low early	1.15	Alberto *et al*. (2011)
	*Q. rubra*	4	Low early	1.93	Mc Gee (1973)
Total				−0.17	
Latitudinal	*P. abies*	9	North early	−2.08	Sogaard *et al*. (2008)
	*P. glauca*	63	No cline	0.43	Li *et al*. (1997a)
	*P. sitchensis*	17	No cline	−0.08	Mimura & Aitken (2007)
	*P. strobus*	66	No cline	−0.83	Li *et al*. (1997b)
	*Populus balsamifera*	4	No cline	0.10	Farmer (1993)
	*Fagus sylvatica*	158	South early	0.20	Von Wuehlisch *et al*. (1995)
	*Q. petraea*	16	South early	4.17	Deans & Harvey (1996)
	*Tsuga heterophylla*	8	South early	2.17	Hannerz *et al*. (1999)
Total				0.51	
(b)					
Altitudinal	*A. lasiocarpa*	5	High early	−3.33	Green (2005)
	*L. occidentalis*	82	High early	−1.28	Rehfeldt (1982)
	*P. abies*	23	High early	−9.07	Chmura (2006)
	*P. abies*	8	High early	−2.63	Skroppa & Magnussen (1993)
	*P. glauca*	5	High early	−1.00	Green (2005)
	*P. contorta*	5	High early	−1.67	Green (2005)
	*P. contorta*	173	High early	−0.22	Rehfeldt (1988)
	*Pseudotsuga menziesii*	7	No cline	0.37	Acevedo-Rodriguez *et al*. (2006)
Total				−2.35	
Latitudinal	*Betula pendula*	7	North early	−4.63	Viherä-Aarnio *et al*. (2005)
	*P. glauca*	63	North early	−3.83	Li *et al*. (1997a)
	*P. sitchensis*	17	North early	−4.90	Mimura & Aitken (2007)
	*P. strobus*	66	North early	−3.33	Li *et al*. (1997b)
	*P. sylvestris*	4	North early	−5.00	Hurme *et al*. (1997)
	*P. sylvestris*	4	North early	−2.35	Notivol *et al*. (2007)
	*P. sylvestris*	2	North early	−6.83	Savolainen *et al*. (2004)
	*P. balsamifera*	4	North early	−5.00	Farmer (1993)
	*P. tremula*	12	North early	−8.33	Luquez *et al*. (2008)
Total				−4.91	

Slopes of linear regressions are given for each study and expressed as days/°C (for details about the calculation see in the text and for references see Table S1). No cline indicates a nonsignificant regression.

* Number of populations in the provenance trial.

In the large set of provenance trial studies, clinal variation along environmental gradients was very common. In the 112 studies, 309 analyses of clinal variation in different quantitative traits, 243 (78%) showed evidence of clinal variation with latitude, altitude, and sometimes longitude (Table S1).

### Adaptation at range margins

An important hypothesis for species range limits is that gene flow constraints adaptation (Haldane, 1932; Mayr, 1963). Many models suggest that gene flow could limit adaptation, and even more so with asymmetrical gene flow toward small peripheral populations (see Lenormand, 2002 for review). In a model of species range involving local adaptation, a strong coupling between fitness and population size favors a feedback effect (a ‘migration meltdown’) that acts to stabilize a range margin, as exemplified in the now well-known Kirkpatrick & Barton (1997) model. However, there is limited evidence to evaluate this model, and some issues that complicate the predictions. Some models assume that genetic variance is fixed (Pease *et al*., 1989; Kirkpatrick & Barton, 1997), while gene flow may also increase genetic variance and the response to selection (Barton, 2001; Polechova *et al*., 2009). Evidence in *P. contorta* suggests that gene flow between populations inhabiting heterogeneous environments can increase levels of standing genetic variation (Yeaman & Jarvis, 2006), but it remains unclear whether this effect would be important in other species. Genetic drift can also reduce genetic variance and thus adaptation in peripheral populations (Alleaume-Benharira *et al*., 2006; Polechova *et al*., 2009; Bridle *et al*., 2010), but gene flow may replenish genetic variation. Gene flow may even introduce better adapted genes than local ones, especially in a changing climate (Alleaume-Benharira *et al*., 2006).

Some environments, in particular some polar or arid range margins, are intrinsically less favorable than others, and would sustain only very low population sizes even after a very long history of adaptation. Mainland-island models of local adaptation implicitly address this issue with population sizes, but spatially continuous models are still more informative. In particular, Nagylaki (1975) showed that extrinsic asymmetries in habitat quality strongly modified or could even compensate for asymmetries in selection across habitats. In other words, alleles showing a local advantage can be maintained despite having considerable antagonistic effects in other habitats, provided that the local habitat is of better quality (Nagylaki, 1978). Incorporating differences in carrying capacity in quantitative models could critically affect the potential for population adaptation (Bridle *et al*., 2010).

The leading and the trailing edge of migrating tree distributions face quite different challenges due to the warming climate (Hampe & Petit, 2005). At the southern range edge (in northern hemisphere), the distributions are likely already limited by high temperatures or drought conditions, and associated biotic and abiotic stresses, whereas at the northern margin, many populations have been limited by the cold temperatures (Rehfeldt *et al*., 2002). For the southern margin, at least at low altitudes, the environment is clearly deteriorating. The risk of extinctions will come from the interplay of multiple factors. In particular, the reduction of water availability and a longer growing season with excessively warm temperatures (IPCC, 2007) could lead to massive diebacks of trees due to drought stress or carbon starvation (Sabate *et al*., 2002; Bréda *et al*., 2006) higher mortality due to reduced defense of trees against insects (Rouault *et al*., 2006), and more frequent forest fires (Mouillot & Field, 2005). Increased mortality due to heat and drought stress has already been observed in many locations globally (Allen *et al*., 2010). The impact of environmental change will be higher in small populations due to high demographic or environmental stochasticity (Hampe & Jump, 2011).

At the southern margin, there are no populations further south contributing genes conferring necessary adaptation, but gene flow from similar environments could still increase the variance within populations (Barton, 2001). Experimental evidence of gene flow from like populations increasing fitness at warm range-edges exists for some plant species (e.g., *Mimulus* species, Sexton *et al*., 2011), and long distance dispersal can be important in fragmented landscapes (Klein *et al*., 2006; Fayard *et al*., 2009; Kremer *et al*., 2012).

Until now, the severe climatic conditions at boreal northern range margins have constrained growth, pollen production, seed maturation and dispersal (Sarvas, 1962; Savolainen, 1996), as well as survival (Persson, 1998), and have limited expansion to the north (Chuine & Beaubien, 2001; Morin *et al*., 2007). In the northernmost areas, temperatures are expected to increase by about 4 °C (Kattsov & Källen, 2005). Ecophysiologists have used the immediate plastic responses of trees to increased temperature to predict changes in species composition (Kellomäki & Kolström, 1992; Kellomäki *et al*., 2001). However, these predictions have not explicitly taken into account the possibilities of genetic response (Davis & Shaw, 2001; O'Neill *et al*., 2008). The warming in the north will improve survival, increase growth (Rehfeldt *et al*., 2002; Reich & Oleksyn, 2008), increase sexual reproduction (Andalo *et al*., 2005), and increase pollen production (Savolainen *et al*., 2011). Based on modeling studies, pollen and seed are predicted to be dispersed further than before (Kuparinen *et al*., 2009, 2010). Production of mature filled seed will likely increase many fold (Kellomäki *et al*., 1997) and the warmer air and soil may result in improved germination and establishment. Northern range margin populations are already colonizing more northern and higher altitude areas (Kullman, 2002; Juntunen *et al*., 2006; Chen *et al*., 2011). The increased survival rates of existing, established trees may, however, reduce establishment opportunities for better adapted genotypes generated by gene flow and local selection (Kuparinen *et al*., 2010).

At altitudinal range limits, adaptation could be facilitated by the short geographical distance between populations, associated with low climate change velocity (Loarie *et al*., 2009). Gene flow from populations at low altitudes could help the populations at higher altitudes to adapt, as has already been observed, e.g., in oak phenological shifts *in situ* (Alberto *et al*., 2010). Both colonization of new areas at higher altitudes, if available, and local selection aided by gene flow may contribute to adaptation, as many altitudinal gradients show clinal genetic differentiation (see above).

## Conclusions and suggestions for future research

Forest trees are exceptionally well characterized with respect to adaptive quantitative variation, and with respect to responses to different climatic variables. The existing set of provenance trials can be used to extract even more information, for instance on the level of local adaptation, or even on the strength of selection, when the datasets are further analyzed. Long-term estimates of the strength of selection, in particular in natural conditions, would be very valuable for providing parameter range estimates for the prediction models. New reciprocal transplant experiments are needed for commercially less-important species, which may be most threatened, but which are under-represented in existing provenance trials. Furthermore, the present provenance trials ignore the likely important early fitness components of germination and establishment – these components also need to be studied (as is being done in herbaceous plants, Huang *et al*., 2010; Stanton-Geddes *et al*., 2012). The new experiments should include field sites at and beyond existing range margins. Experiments in controlled growth chambers can also help identify those abiotic aspects of temperature and moisture regimes to which populations are locally adapted, and to generate climatic regimes analogous to those predicted for the coming century.

The role of plasticity and its interaction with natural selection is just starting to be explored in the climate change context (Chevin *et al*., 2010) – provenance trials can also provide more information on these aspects. The extent and significance of adaptive phenotypic plasticity is still debated (Valladares *et al*., 2007), and experimental studies on range margins are still few (Angert, 2009; Stanton-Geddes *et al*., 2012). Wang *et al*. (2010) universal response function approach could be used as a mechanistic model to predict population responses.

Commercially less-important species are poorly represented in previously established common gardens, whether they have narrow or wide distributions. The species with smaller ranges are especially vulnerable. Are these species locally adapted to climate? Do these species have limited adaptive potential due to their historically small effective population sizes? While many important boreal and temperate species in the northern hemisphere (and some eucalypts or tropical acacias) have been extensively studied, there is much less information on subtropical or tropical species, which are outside the scope of this review. These species will also be affected by the changing climate, through both abiotic and many complex biotic factors.

Most of the studies on quantitative traits have been conducted in spaced, reasonably well-tended provenance trial experiments. Within or between-species interactions, such as competition or diseases have largely been ignored. Many between-species interactions depend on the synchronous timing of events in the different species. Even before any evolutionary responses, phenotypic responses will affect such biotic interactions (Gilman *et al*., 2010; Yang & Rudolf, 2010). During the past decade, phenological shifts have been already observed between trees and pest populations (Visser & Holleman, 2001; van Asch *et al*., 2007; Desprez-Loustau *et al*., 2010; Gordo & Sanz, 2010).

Much of the information on northern trees has been accumulated through decades of field experiments. Combining genomic tools with results from the quantitative and ecological approaches can significantly aid in predicting selection responses to climate change (for crop plants, see Morrell *et al*., 2012). Genomic studies will allow researchers to examine the geographical pattern of alleles conferring adaptation – are they globally occurring alleles with varying frequencies or very localized ones? Coupled with studies at the quantitative trait level, genomic surveys will aid in assessing the prospects for adaptation at the level of the population. Furthermore, the contribution of epigenetic and maternal effects to phenotypic variation needs to be assessed.

This review has pointed to several areas where management and breeding can possibly contribute to maintenance of populations. An evaluation of such options is beyond the scope of this review (see e.g., McLachlan *et al*., 2007; Aitken & Whitlock, 2013).

In conclusion, the concordant patterns of current local adaptation among tree populations in numerous northern species in Europe and North America show that selection has repeatedly established such patterns. Populations facing the largest evolutionary challenges are at the range margins, but the northern and southern margins face quite different limitations. Better data and models are thus necessary to evaluate accurately whether natural selection, and migration, may again allow evolutionary responses for populations to sufficiently match their new climates.

## Supporting Information

**Table S1.** Description of the provenance trial studies. ^a^Number of populations sampled. ^b^Mean number of individuals sampled within populations. Bulk indicates that mixes of seeds without knowledge of genetic identity were collected in the populations. ^c^Number of provenance trials in the study. ^d^Amplitude of the environmental gradient is given in meters of elevation above sea level for altitudinal gradients and in decimal degrees for latitudinal and longitudinal gradients. When values appear in italic the approximate amplitude is given. ^e^The levels of population differentiation in the provenance trials were measured either as the proportion of the total phenotypic variation which is between populations (*V*_pop_) or as the proportion of the additive genetic variance which is between populations (*Q*_ST_), which appear in bold. For simplicity, we used *Q*_ST_ for both parameters in the text. No differentiation indicates that population differentiation was not significant. ^f^Slopes were calculated for bud flush and bud set only because they were the only traits with enough data to make comparisons between traits and between environmental gradients. No cline indicates that clinal variation was not significant along the environmental gradient considered. *n.i*. means that the information was not indicated.

**Table S2.** Nucleotide diversity estimates per gene. ^a^π_total_: Nucleotide diversity per gene calculated for silent and replacement sites. ^b^π_silent_: Nucleotide diversity per gene calculated for silent sites only, which correspond to synonymous sites and sites located in noncoding regions (introns or 3′- and 5′-UTR) *n.i*. means that the value was not indicated. Cells highlighted in gray indicate studies for which average nucleotide diversity were calculated for a set of genes or fragments of genes.

**Table S3.** SNP effect sizes in association studies. ^a^R² marker: Percentage of phenotypic variance explained by the marker.

## References

[b1] Acevedo-Rodriguez R, Vargas-Hernandez JJ, Lopez-Upton J, Mendoza JV (2006). Effect of geographic origin and nutrition on shoot prenology of Mexican Douglas-Fir (Pseudotsuga sp.) seedlings. Agrociencia.

[b2] Aitken SN, Whitlock MC (2013). Assisted gene flow to facilitate local adaptation to climate change. Annual Review of Ecology, Evolution, and Systematics.

[b3] Aitken SN, Yeaman S, Holliday JA, Wang TL, Curtis-McLane S (2008). Adaptation, migration or extirpation: climate change outcomes for tree populations. Evolutionary Applications.

[b4] Alberto F, Niort J, Derory J, Lepais O, Vitalis R, Galop D, Kremer A (2010). Population differentiation of sessile oak at the altitudinal front of migration in the French Pyrenees. Molecular Ecology.

[b5] Alberto F, Bouffier L, Louvet JM, Lamy JB, Delzon S, Kremer A (2011). Adaptive responses for seed and leaf phenology in natural populations of sessile oak along an altitudinal gradient. Journal of Evolutionary Biology.

[b6] Alleaume-Benharira M, Pen IR, Ronce O (2006). Geographical patterns of adaptation within a species’ range: interactions between drift and gene flow. Journal of Evolutionary Biology.

[b7] Allen CD, Macalady AK, Chenchouni H (2010). A global overview of drought and heat-induced tree mortality reveals emerging climate change risks for forests. Forest Ecology and Management.

[b8] Andalo C, Beaulieu J, Bousquet J (2005). The impact of climate change on growth of local white spruce populations in Quebec. Forest Ecology and Management.

[b9] Angert AL (2009). The niche, limits to species’ distributions, and spatiotemporal variation in demography across the elevation ranges of two monkeyflowers. Proceedings of the National Academy of Sciences.

[b10] van Asch M, Visser ME (2007). Phenology of forest caterpillars and their host trees: the importance of synchrony. Annual Review of Entomology.

[b11] van Asch M, Tienderen PH, Holleman LJM, Visser ME (2007). Predicting adaptation of phenology in response to climate change, an insect herbivore example. Global Change Biology.

[b12] Atwell S, Huang YS, Vilhjalmsson BJ (2010). Genome-wide association study of 107 phenotypes in *Arabidopsis thaliana* inbred lines. Nature.

[b13] Barton NH (1999). Clines in polygenic traits. Genetical Research.

[b14] Barton N, Silvertown J, Antonovics J (2001). Adaptation at the edge of a species’ range. Integrating Genetics and Ecology an A Spatial Context.

[b15] Barton NH, Keightley PD (2002). Understanding quantitative genetic variation. Nature Reviews Genetics.

[b16] Basler D, Körner C (2012). Photoperiod sensitivity of bud burst in 14 temperate forest tree species. Agricultural and Forest Meteorology.

[b17] Benito-Garzón M, Alia R, Robson TM, Zavala MA (2011). Intra-specific variability and plasticity influence potential tree species distributions under climate change. Global Ecology and Biogeography.

[b18] Beuker E, Valtonen E, Repo T (1998). Seasonal variation in the frost hardiness of Scots pine and Norway spruce in old provenance experiments in Finland. Forest Ecology and Management.

[b19] Björklund M, Ranta E, Kaitala V, Bach LA, Lundberg P, Stenseth NC (2009). Quantitative trait evolution and environmental change. PLoS ONE.

[b20] Böhlenius H, Huang T, Charbonnel-Campaa L, Brunner AM, Jansson S, Strauss SH, Nilsson O (2006). CO/FT regulatory module controls timing of flowering and seasonal growth cessation in trees. Science.

[b21] Bräutigam K, Vining KJ, Lafon-Placette C (2013). Epigenetic regulation of adaptive responses of forest tree species to the environment. Ecology and Evolution.

[b22] Bréda N, Huc R, Granier A, Dreyer E (2006). Temperate forest trees and stands under severe drought: a review of ecophysiological responses, adaptation processes and long-term consequences. Annals of Forest Science.

[b23] Bridle JR, Polechova J, Kawata M, Butlin RK (2010). Why is adaptation prevented at ecological margins? New insights from individual-based simulations. Ecology Letters.

[b24] Buckler ES, Holland JB, Bradbury PJ (2009). The Genetic Architecture of Maize Flowering Time. Science.

[b25] Burger R, Lynch M (1995). Evolution and extinction in a changing environments - a quantitative genetic analysis. Evolution.

[b26] Caffarra A, Donelly A, Chuine I (2011). Modelling the timing of Betula pubescens budburst. II. Integrating the complex effects of photoperiod into process-based models of budburst. Climate Research.

[b27] Charlesworth B, Charlesworth D (2010). Elements of Evolutionary Genetics.

[b28] Cheddadi R, Vendramin GG, Litt T (2006). Imprints of glacial refugia in the modern genetic diversity of *Pinus sylvestris*. Global Ecology and Biogeography.

[b29] Chen J, Källman T, Gyllenstrand N, Lascoux M (2009). New insights on the speciation history and nucleotide diversity of three boreal spruce species and a Tertiary relict. Heredity.

[b30] Chen IC, Hill JK, Ohlemuller R, Roy DB, Thomas CD (2011). Rapid range shifts of species associated with high levels of climate warming. Science.

[b31] Chevin L-M, Lande R (2010). When do adaptive plasticity and genetic evolution prevent extinction of a density-regulated population?. Evolution.

[b32] Chevin L-M, Lande R, Mace GM (2010). Adaptation, plasticity, and extinction in a changing environment: towards a predictive theory. PLoS Biology.

[b33] Chmura DJ (2006). Phenology differs among Norway spruce populations in relation to local variation in altitude of maternal stands in the Beskidy Mountains. New Forests.

[b34] Chuine I, Beaubien EG (2001). Phenology is a major determinant of tree species range. Ecology Letters.

[b35] Conner JK, Franks R, Stewart C (2003). Expression of additive genetic variances and covariances for wild radish floral traits: comparison between field and greenhouse environments. Evolution.

[b36] Cornelius J (1994). Heritabilities and additive genetic coefficients of variation in forest trees. Canadian Journal of Forest. Research.

[b37] Cumbie WP, Eckert AJ, Wegrzyn JL, Whetten R, Neale DB, Goldfarb B (2011). Association genetics of carbon isotope discrimination, height, and foliar nitrogen in a natural population of *Pinus taeda* L. Heredity.

[b38] Davis MB, Shaw RG (2001). Range shifts and adaptive responses to quaternary climate changes. Science.

[b39] De Carvalho D, Ingvarsson PK, Joseph J (2010). Admixture facilitates adaptation from standing variation in the European aspen (*Populus tremula* L.), a widespread forest tree. Molecular Ecology.

[b40] Deans JD, Harvey FJ (1996). Frost hardiness of 16 European provenances of sessile oak growing in Scotland. Forestry.

[b41] Desprez-Loustau ML, Vitasse Y, Delzon S, Capdevielle X, Marcais B, Kremer A (2010). Are plant pathogen populations adapted for encounter with their host? A case study of phenological synchrony between oak and an obligate fungal parasite along an altitudinal gradient. Journal of Evolutionary Biology.

[b42] Duputie A, Massol F, Chuine I, Kirkpatrick M, Ronce O (2012). How do genetic correlations affect species range shifts in a changing environment?. Ecology Letters.

[b43] Eckert AJ, Bower AD, Wegrzyn JL (2009). Asssociation genetics of Coastal Douglas Fir (*Pseudotsuga menziesii* var. *menziesii,* Pinaceae). I. Cold-fardiness related traits. Genetics.

[b44] Eckert AJ, van Heerwaarden J, Wegrzyn JL, Nelson CD, Ross-Ibarra J, Gonzalez-Martinez SC, Neale DB (2010). Patterns of population structure and environmental associations to aridity across the range of Loblolly Pine (*Pinus taeda* L., Pinaceae). Genetics.

[b45] Edelaar P, Burraco P, Gomez-Mestre I (2011). Comparisons between Q(ST) and F(ST)-how wrong have we been?. Molecular Ecology.

[b46] Etterson JR, Shaw RG (2001). Constraint to adaptive evolution in response to global warming. Science.

[b47] Falconer DS, Mackay TFC (1996). Introduction to Quantitative Genetics.

[b48] Farmer RE (1993). Latitudinal variation in height and phenology of balsam poplar. Silvae Genetica.

[b49] Fayard J, Klein EK, Lefevre F (2009). Long distance dispersal and the fate of a gene from the colonization front. Journal of Evolutionary Biology.

[b50] Fisher RA (1918). The correlation between relatives on the supposition of Mendelian inheritance. Transactions of the Royal Society of Edinburgh.

[b51] Fisher RA (1930). The Genetical Theory of Natural Selection.

[b52] Franks SJ, Hoffmann AA (2012). Genetics of Climate Change Adaptation. Annual Review of Genetics.

[b53] Gienapp P, Teplitsky C, Alho JS, Mills JA, Merilä J (2008). Climate change and evolution: disentangling environmental and genetic responses. Molecular Ecology.

[b54] Gilman SE, Urban MC, Tewksbury J, Gilchrist GW, Holt RD (2010). A framework for community interactions under climate change. Trends in Ecology & Evolution.

[b55] Goldstein DB, Holsinger KE (1992). Maintenance of polygenic variation in spatially structured populations: roles for local mating and genetic redundancy. Evolution.

[b56] Gomulkiewicz R, Holt RD (1995). When does evolution by natural selection prevent extinction. Evolution.

[b57] Gomulkiewicz R, Houle D (2009). Demographic and genetic constraints on evolution. The American naturalist.

[b58] Gomulkiewicz R, Holt RD, Barfield M, Nuismer SL (2010). Genetics, adaptation, and invasion in harsh environments. Evolutionary Applications.

[b59] Gordo O, Sanz JJ (2010). Impact of climate change on plant phenology in Mediterranean ecosystems. Global Change Biology.

[b60] Grattapaglia D, Resende MDV (2011). Genomic selection in forest tree breeding. Tree Genetics & Genomes.

[b61] Green DS (2005). Adaptive strategies in seedlings of three co-occurring, ecologically distinct northern coniferous tree species across an elevational gradient. Canadian Journal of Forest Research.

[b62] Grivet D, Sebastiani F, Alía R (2011). Molecular footprints of local adaptation in two Mediterranean conifers. Molecular Biology and Evolution.

[b63] Haldane JBS (1932). The Causes of Evolution.

[b64] Hampe A, Jump AS (2011). Climate relicts: past, present, future. Annual Review of Ecology, Evolution, and Systematics.

[b65] Hampe A, Petit RJ (2005). Conserving biodiversity under climate change: the rear edge matters. Ecology Letters.

[b66] Hancock AM, Brachi B, Faure N (2011). Adaptation to climate across the *Arabidopsis thaliana* genome. Science.

[b67] Hannerz M, Aitken SN, King JN, Budge S (1999). Effects of genetic selection for growth on frost hardiness in western hemlock. Canadian Journal of Forest Research-Revue Canadienne De Recherche Forestiere.

[b68] Hänninen H, Tanino K (2011). Tree seasonality in a warming climate. Trends in Plant Science.

[b69] Hedrick PW (2006). Genetic polymorphism in heterogeneous environments: the age of genomics. Annual Review of Ecology Evolution and Systematics.

[b70] Hendry AP, Kinnison MT (1999). Perspective: the pace of modern life: measuring rates of contemporary microevolution. Evolution.

[b71] Hendry AP, Day T, Taylor EB (2001). Population mixing and the adaptive divergence of quantitative traits in discrete populations: a theoretical framework for empirical tests. Evolution.

[b72] Heuertz M, De Paoli E, Källman T (2006). Multilocus patterns of nucleotide diversity, linkage disequilibrium and demographic history of Norway spruce (*Picea abies* (L.) Karst). Genetics.

[b73] Hill WG, Goddard ME, Visscher PM (2008). Data and theory point to mainly additive genetic variance for complex traits. PLoS Genetics.

[b74] Hill JK, Griffiths HM, Thomas CD (2011). Climate change and evolutionary adaptations at species’ range margins. Annual Review of Entomology.

[b75] Hoffmann AA, Sgrò CM (2011). Climate change and evolutionary adaptation. Nature.

[b76] Holliday JA, Ritland K, Aitken SN (2010a). Widespread, ecologically relevant genetic markers developed from association mapping of climate-related traits in Sitka spruce (*Picea sitchensis*. New Phytologist.

[b77] Holliday JA, Yuen M, Ritland K, Aitken SN (2010b). Postglacial history of a widespread conifer produces inverse clines in selective neutrality tests. Molecular Ecology.

[b78] Holliday JA, Wang T, Aitken S (2012). Predicting Adaptive Phenotypes From Multilocus Genotypes in Sitka Spruce (Picea sitchensis) Using Random Forest. G3. Genes Genomes Genetics.

[b79] Howe GT, Aitken SN, Neale DB, Jermstad KD, Wheeler NC, Chen THH (2003). From genotype to phenotype: unraveling the complexities of cold adaptation in forest trees. Canadian Journal of Botany.

[b80] Huang X, Schmitt J, Dorn L (2010). The earliest stages of adaptation in an experimental plant population: strong selection on QTLS for seed dormancy. Molecular Ecology.

[b81] Hurme P, Repo T, Pääkkönen T, Savolainen O (1997). Climatic adaptation of bud set and frost hardiness in Scots pine (*Pinus sylvestris* L.). Canadian Journal of Forest Research.

[b82] Ingvarsson PK, Garcia MV, Luquez V, Hall D, Jansson S (2008). Nucleotide polymorphism and phenotypic associations within and around the phytochrome B2 locus in European aspen (*Populus tremula*, Salicaceae). Genetics.

[b83] Houghton JT, Ding Y, Griggs DJ, Noguer M, Van DerLindenPJ, Dai X, Maskell K, Johnsson CA, IPCC (2001). The Scientific Basis. Climate Change 2001.

[b84] Parry ML, Canziani OF, Palutikof JP, van der Linden PJ, Hanson CE, IPCC (2007). Contribution of Working Group II to the Fourth Assessment Report of the Intergovernmental Panel on Climate Change. Climate Change 2007: Impacts, Adaptation and Vulnerability.

[b85] Iwata H, Hayashi T, Tsumura Y (2011). Prospects for genomic selection in conifer breeding: a simulation study of *Cryptomeria japonica*. Tree Genetics Genomes.

[b86] Jalas J, Suominen T (1973). Atlas Floreae Europaea. Coniferales.

[b87] Jermstad KD, Bassoni DL, Jech KS, Ritchie GA, Wheeler NC, Neale DB (2003). Mapping of quantitative trait loci controlling adaptive traits in coastal Douglas Fir. III. Quantitative trait loci-by-environment interactions. Genetics.

[b88] Johannes F, Porcher E, Teixeira FK (2009). Assessing the impact of transgenerational epigenetic variation on complex traits. PLoS Genetics.

[b89] Jump AS, Matyas C, Penuelas J (2009). The altitude-for-latitude disparity in the range retractions of woody species. Trends in Ecology & Evolution.

[b90] Juntunen V, Neuvonen S, Sutinen R (2006). Männyn puurajan muutokset viimeisen 400 vuoden aikana ja metsänraja-puuraja vaihettumisvyöhykkeen ikärakenne. Metlan työraportteja.

[b91] Kattsov VM, Källen E, Simon C, Arres L, Heal B (2005). Future climate change: modeling and scenarios for the Arctic. Arctic Climate Impact Assessment - scientific report.

[b92] Kawecki TJ, Ebert D (2004). Conceptual issues in local adaptation. Ecology Letters.

[b93] Kayihan GC, Nelson CD, Huber DA, Amerson HV, White TL, Davis JM (2010). Clonal evaluation for fusiform rust disease resistance: effects of pathogen virulence and disease escape. Canadian Journal of Forest Research.

[b94] Kellomäki S, Kolström M (1992). Simulation of tree species composition and organic matter accumulation in Finnish boreal forests under changing climatic conditions. Vegetatio.

[b95] Kellomäki S, Väisänen H, Kolström T (1997). Model computatons on the effects of elevating temperature and atmosphere CO2 on the regeneration of Scots pine at te timber line in Finland. Climatic Change.

[b96] Kellomäki S, Rouvinen I, Peltola H, Strandman H, Steinbrecher R (2001). Impact of global warming on the tree species composition of boreal forests in Finland and effects on emission of isoprenoids. Global Change Biology.

[b97] Kingsolver JG, Diamond SE (2011). Phenotypic selection in natural populations: what limits directional selection?. The American naturalist.

[b98] Kingsolver JG, Hoekstra HE, Hoekstra JM (2001). The strength of phenotypic selection in natural populations. The American naturalist.

[b99] Kirkpatrick M, Barton NH (1997). Evolution of a species’ range. The American naturalist.

[b100] Klein EK, Lavigne C, Gouyon PH (2006). Mixing of propagules from discrete sources at long distance: comparing a dispersal tail to an exponential. BMC Ecology.

[b101] Körner C, Basler D (2010). Phenology under global warming. Science.

[b102] Kozlowski T, Pallardy S (2002). Acclimation and adaptive responses of woody plants to environmental stresses. The Botanical Review.

[b103] Kramer K (1995). Phenotypic plasticity of the phenology of 7 European tree species in relation to climate warming. Plant Cell and Environment.

[b104] Kremer A, Le Corre V (2012). Decoupling of differentiation between traits and their underlying genes in response to divergent selection. Heredity.

[b105] Kremer A, Ronce O, Robledo-Arnuncio JJ (2012). Long-distance gene flow and adaptation of forest trees to rapid climate change. Ecology Letters.

[b106] Kullman L (2002). Rapid recent range-margin rise of tree and shrub species in the Swedish Scandes. Journal of Ecology.

[b107] Kuparinen A, Katul G, Nathan R, Schurr FM (2009). Increases in air temperature can promote wind-driven dispersal and spread of plants. Proceedings. Biological sciences/The Royal Society.

[b108] Kuparinen A, Savolainen O, Schurr FM (2010). Increased mortality can promote evolutionary adaptation of forest trees to climate change. Forest Ecology and Management.

[b109] Lande R (2009). Adaptation to an extraordinary environment by evolution of phenotypic plasticity and genetic assimilation. Journal of Evolutionary Biology.

[b110] Langlet O (1971). Two hundred years of genecology. Taxon.

[b111] Latta RG (1998). Differentiation of allelic frequencies at quantitative trait loci affecting locally adaptive traits. The American naturalist.

[b112] Laurie CC, Chasalow SD, LeDeaux JR (2004). The genetic architecture of response to long-term artificial selection for oil concentration in the maize kernel. Genetics.

[b113] Le Corre V, Kremer A (2012). The genetic differentiation at quantitative trait loci under local adaptation. Molecular Ecology.

[b114] LeCorre V, Kremer A (2003). Genetic variability at neutral markers, quantitative trait loci and trait in a subdivided population under selection. Genetics.

[b115] Leimu R, Fischer M (2008). A meta-analysis of local adaptation in plants. PLoS ONE.

[b116] Leinonen T, O'Hara RB, Cano JM, Merila J (2008). Comparative studies of quantitative trait and neutral marker divergence: a meta-analysis. Journal of Evolutionary Biology.

[b117] Lenormand T (2002). Gene flow and the limits to natural selection. Trends in Ecology & Evolution.

[b118] Li P, Beaulieu J, Bousquet J (1997a). Genetic structure and patterns of genetic variation among populations in eastern white spruce. Canadian Journal of Forest Research.

[b119] Li P, Beaulieu J, Daoust G, Plourde A (1997b). Patterns of adaptive genetic variation in eastern white pine (*Pinus strobus*) from Quebec. Canadian Journal of Forest Research-Revue Canadienne De Recherche Forestiere.

[b120] Lindner M, Maroschek M, Netherer S (2010). Climate change impacts, adaptive capacity, and vulnerability of European forest ecosystems. Forest Ecology and Management.

[b121] Loarie SR, Duffy PB, Hamilton H, Asner GP, Field CB, Ackerly DD (2009). The velocity of climate change. Nature.

[b122] Luquez V, Hall D, Albrectsen BR, Karlsson J, Ingvarsson P, Jansson S (2008). Natural phenological variation in aspen (Populus tremula): the SwAsp collection. Tree Genetics & Genomes.

[b123] Lynch M, Avise JC, Hamrick JL (1996). A quantitative-genetic perspective on conservation issues. Conservation Genetics. Case Histories From Nature.

[b124] Lynch M, Lande R, Kareiva PM, Kingsolver JG, Huey RB (1993). Evolution and extinction in response to environmental change. Biotic Interactions and Global Change.

[b125] Magri D, Vendramin GG, Comps B (2006). A new scenario for the Quaternary history of European beech populations: palaeobotanical evidence and genetic consequences. New Phytologist.

[b126] Mayr E (1963). Animal Species and Evolution.

[b127] Mc Gee CE (1973). Is variation in budbreak of Red oak the result of heredity or environment?. Proceeding of the 12th Southern Forest Tree Improvement Conference.

[b128] McLachlan JS, Clark JS, Manos PS (2005). Molecular indicators of tree migration capacity under rapid climate change. Ecology.

[b129] McLachlan JS, Hellmann JJ, Schwartz MW (2007). A framework for debate of assisted migration in an era of climate change. Conservation Biology.

[b130] Menzel A, Fabian P (1999). Growing season extended in Europe. Nature.

[b131] Menzel A, Sparks TH, Estrella N (2006). European phenological response to climate change matches the warming pattern. Global Change Biology.

[b132] Mikola J (1982). Bud-set phenology as an indicator of climatic adaptation of Scots pine in Finland. Silva Fennica.

[b133] Mimura M, Aitken SN (2007). Adaptive gradients and isolation-by-distance with postglacial migration in *Picea sitchensis*. Heredity.

[b134] Morgenstern EK (1978). Range-wide variation of black spruce. Canadian Journal of Forest Research.

[b135] Morgenstern EK (1996). Geographic Variation in Forest Trees: Genetic Basis and Application of Knowledge in Silviculture.

[b136] Morin X, Thuiller W (2009). Comparing niche- and process-based models to reduce prediction uncertainty in species range shifts under climate change. Ecology.

[b137] Morin X, Augspurger C, Chuine I (2007). Process-based modeling of species’ distributions: what limits temperate tree species’ range boundaries?. Ecology.

[b138] Morrell PL, Buckler ES, Ross-Ibarra J (2012). Crop genomics: advances and applications. Nature Reviews Genetics.

[b139] Mouillot F, Field CB (2005). Fire history and the global carbon budget: a 1 degrees x 1 degrees fire history reconstruction for the 20th century. Global Change Biology.

[b140] Nagylaki T (1975). Conditions for existence of clines. Genetics.

[b141] Nagylaki T (1978). Clines with asymmetric migration. Genetics.

[b142] Namroud MC, Beaulieu J, Juge N, Laroche J, Bousquet J (2008). Scanning the genome for gene single nucleotide polymorphisms involved in adaptive population differentiation in white spruce. Molecular Ecology.

[b143] Neale DB, Kremer A (2011). Forest tree genomics: growing resources and applications. Nature Reviews Genetics.

[b144] Netherer S, Schopf A (2010). Potential effects of climate change on insect herbivores in European forests-General aspects and the pine processionary moth as specific example. Forest Ecology and Management.

[b145] Nicotra AB, Atkin OK, Bonser SP (2010). Plant phenotypic plasticity in a changing climate. Trends in Plant Science.

[b146] Niinemets U (2010). Responses of forest trees to single and multiple environmental stresses from seedlings to mature plants: past stress history, stress interactions, tolerance and acclimation. Forest Ecology and Management.

[b147] Notivol E, Garcia-Gil MR, Alia R, Savolainen O (2007). Genetic variation of growth rhythm traits in the limits of a latitudinal cline in Scots pine. Canadian Journal of Forest Research-Revue Canadienne De Recherche Forestiere.

[b148] Olson-Manning CF, Wagner MR, Mitchell-Olds T (2012). Adaptive evolution: evaluating empirical support for theoretical predictions. Nature Reviews Genetics.

[b149] O'Neill GA, Hamann A, Wang TL (2008). Accounting for population variation improves estimates of the impact of climate change on species’ growth and distribution. Journal of Applied Ecology.

[b150] Orr HA (1998). The population genetics of adaptation: the distribution of factors fixed during adaptive evolution. Evolution.

[b151] Parducci L, Jorgensen T, Tollefsrud MM (2012). Glacial survival of boreal trees in Northern Scandinavia. Science.

[b152] Parmesan C (2006). Ecological and evolutionary responses to recent climate change. Annual Review of Ecology, Evolution and Systematics.

[b153] Pease CM, Lande R, Bull JJ (1989). A model of population growth, dispersal and evolution in a changing environment. Ecology.

[b154] Persson B (1998). Will climate change affect the optimal choice of *Pinus sylvestris* provenances. Silva Fennica.

[b155] Petit RJ, Hampe A (2006). Some evolutionary consequences of being a tree. Annual Review of Ecology Evolution and Systematics.

[b156] Petit RJ, Brewer S, Bordacs S (2002). Identification of refugia and post-glacial colonisation routes of European white oaks based on chloroplast DNA and fossil pollen evidence. Forest Ecology and Management.

[b157] Polechova J, Barton N, Marion G (2009). Species’ range: adaptation in space and time. The American naturalist.

[b158] Pyhäjärvi T, García-Gil MR, Knürr T, Mikkonen M, Wachowiak W, Savolainen O (2007). Demographic history has influenced nucleotide diversity in European *Pinus sylvestris* populations. Genetics.

[b159] Quesada T, Gopal V, Cumbie WP (2010). Association mapping of quantitative disease resistance in a natural population of Loblolly Pine (*Pinus taeda* L.). Genetics.

[b160] Rehfeldt GE (1978). Genetic differentiation of Douglas fir populations from the northern Rocky Mountains. Ecology.

[b161] Rehfeldt GE (1982). Differentiation of *Larix occidentalis* populations from the northern Rocky Mountains. Silvae Genetica.

[b162] Rehfeldt GE (1988). Ecological genetics of *Pinus contorta* from the Rocky mountains (USA): a synthesis. Silvae Genet.

[b163] Rehfeldt GE, Jaquish BC (2010). Ecological impacts and management strategies for western larch in the face of climate-change. Mitigation and Adaptation Strategies for Global Change.

[b164] Rehfeldt GE, Ying CC, Spittlehouse DL, Hamilton DA (1999). Genetic responses to climate change in *Pinus contorta*: niche breadth, climate change, and reforestation. Ecological Monographs.

[b165] Rehfeldt GE, Wykoff WR, Ying CC (2001). Physiologic plasticity, evolution, and impacts of a changing climate on Pinus contorta. Climatic Change.

[b166] Rehfeldt GE, Tchebakova NM, Parfenova YI, Wykoff WR, Kuzmina NA, Milyutin LI (2002). Intraspecific responses to climate in *Pinus sylvestris*. Global Change Biology.

[b167] Reich PB, Oleksyn J (2008). Climate warming will reduce growth and survival of Scots pine except in the far north. Ecology Letters.

[b168] Resende MFR, Munoz P, Acosta JJ (2012). Accelerating the domestication of trees using genomic selection: accuracy of prediction models across ages and environments. New Phytologist.

[b169] Richards CL, Bossdorf O, Verhoeven KJF (2010). Understanding natural epigenetic variation. New Phytologist.

[b170] Ritland K (1996). Marker-based method for inferences about quantitative inheritance in natural populations. Evolution.

[b171] Robertson C, Nelson TA, Jelinski DE, Wulder MA, Boots B (2009). Spatial-temporal analysis of species range expansion: the case of the mountain pine beetle, Dendroctonus ponderosae. Journal of Biogeography.

[b172] Rohde A, Bhalerao RP (2007). Plant dormancy in the perennial context. Trends in Plant Science.

[b173] Rouault G, Candau JN, Lieutier F, Nageleisen LM, Martin JC, Warzee N (2006). Effects of drought and heat on forest insect populations in relation to the 2003 drought in Western Europe. Annals of Forest Science.

[b174] Sabate S, Gracia CA, Sanchez A (2002). Likely effects of climate change on growth of Quercus ilex, Pinus halepensis, Pinus pinaster, Pinus sylvestris and Fagus sylvatica forests in the Mediterranean region. Forest Ecology and Management.

[b175] Sarvas R (1962). Investigations on the flowering and seed crop of *Pinus sylvestris*. Communicationes Instituti Forestalia Fennica.

[b176] Savolainen O (1996). Pines beyond the polar circle: adaptation to stress conditions. Euphytica.

[b177] Savolainen O, Pyhäjärvi T (2007). Genomic diversity in forest trees. Current Opinion in Plant Biology.

[b178] Savolainen O, Bokma F, Garcia-Gil R, Komulainen P, Repo T (2004). Genetic variation in cessation of growth and frost hardiness and consequences for adaptation of Pinus sylvestris to climatic changes. Forest Ecology and Management.

[b179] Savolainen O, Pyhajarvi T, Knurr T (2007). Gene flow and local adaptation in trees. Annual Review of Ecology Evolution and Systematics.

[b180] Savolainen O, Kujala ST, Sokol C (2011). Adaptive potential of northernmost tree populations to climate change, with emphasis on Scots pine (*Pinus sylvestris* L.). Journal of Heredity.

[b181] Sexton JP, Strauss SY, Rice KJ (2011). Gene flow increases fitness at the warm edge of a species’ range. Proceedings of the National Academy of Sciences.

[b182] Shaw RG, Etterson JR (2012). Rapid climate change and the rate of adaptation: insight from experimental quantitative genetics. New Phytologist.

[b183] Shutyaev AM, Giertych M (1997). Height growth variation in a comprehensive Eurasian provenance experiment of *Pinus sylvestris* L. Silvae Genetica.

[b184] Sillanpää MJ (2011). On statistical methods for estimating heritability in wild populations. Molecular Ecology.

[b185] Skrøppa T, Kohmann K (1997). Adaptation to local conditions after one generation in Norway spruce. Forest Genetics.

[b186] Skroppa T, Magnussen S (1993). Provenance variation in shoot growth components of Norway spruce. Silvae Genetica.

[b187] Sogaard G, Johnsen O, Nilsen J, Junttila O (2008). Climatic control of bud burst in young seedlings of nine provenances of Norway spruce. Tree Physiology.

[b188] Soltis DE, Morris AB, McLachlan JS, Manos PS, Soltis PS (2006). Comparative phylogeography of unglaciated eastern North America. Molecular Ecology.

[b189] Soto A, Robledo-Arnuncio JJ, González-Martínez SC, Smouse PE, Alía R (2010). Climatic niche and neutral genetic diversity of the six Iberian pine species: a retrospective and prospective view. Molecular Ecology.

[b190] Stanton-Geddes J, Shaw RG, Tiffin P (2012). Interactions between soil habitat and geographic range location affect plant fitness. PLoS ONE.

[b191] Sundblad LG, Andersson B (1995). No difference in frost hardiness between high and low altitude Pinus sylvestris (L) offspring. Scandinavian Journal of Forest Research.

[b192] Tian F, Bradbury PJ, Brown PJ (2011). Genome-wide association study of leaf architecture in the maize nested association mapping population. Nature Genetics.

[b193] Turchin MC, Chiang CWK, Palmer CD, Sankararaman S, Reich D, Hirschhorn JN, Genetic Invest A (2012). Evidence of widespread selection on standing variation in Europe at height-associated SNPs. Nature Genetics.

[b194] Valladares F, Gianoli E, Gomez JM (2007). Ecological limits to plant phenotypic plasticity. New Phytologist.

[b195] Vendramin GG, Fady B, Gonzalez-Martinez SC (2008). Genetically depauperate but widespread: the case of an emblematic mediterranean pine. Evolution.

[b196] Viherä-Aarnio A, Hakkinen R, Partanen J, Luomajoki A, Koski V (2005). Effects of seed origin and sowing time on timing of height growth cessation of Betula pendula seedlings. Tree Physiology.

[b197] Visser ME, Holleman LJM (2001). Warmer springs disrupt the synchrony of oak and winter moth phenology. Proceedings of the Royal Society of London Series B-Biological Sciences.

[b198] Vitasse Y, Delzon S, Bresson CC, Michalet R, Kremer A (2009). Altitudinal differentiation in growth and phenology among populations of temperate-zone tree species growing in a common garden. Canadian Journal of Forest Research.

[b199] Vitasse Y, Bresson CC, Kremer A, Michalet R, Delzon S (2010). Quantifying phenological plasticity to temperature in two temperate tree species. Functional Ecology.

[b200] Voltas J, Chambel M, Prada M, Ferrio J (2008). Climate-related variability in carbon and oxygen stable isotopes among populations of Aleppo pine grown in common-garden tests. Trees-Structure and Function.

[b201] Von Wuehlisch G, Krusche D, Muhs HJ (1995). Variation in temperature sum requirement for flushing of beech provenances. Silvae Genetica.

[b202] Wang T, Hamann A, Yanchuk A, O'Neill GA, Aitken SN (2006). Use of response functions in selecting lodgepole pine populations for future climates. Global Change Biology.

[b203] Wang TL, O'Neill GA, Aitken SN (2010). Integrating environmental and genetic effects to predict responses of tree populations to climate. Ecological Applications.

[b204] Wilczek AM, Burghardt LT, Cobb AR, Cooper MD, Welch SM, Schmitt J (2010). Genetic and physiological bases for phenological responses to current and predicted climates. Philosophical transactions of the Royal Society of London. Series B, Biological sciences.

[b205] Worrall J (1983). Temperature - bud-burst relationships in Amabilis and Subalpine fir provenance tests replicated at different elevations. Silvae Genetica.

[b206] Worrell R, Cundall EP, Malcolm DC, Ennos RA (2000). Variation among seed sources of silver birch in Scotland. Forestry.

[b207] Wright S (1931). Evolution in Mendelian populations. Genetics.

[b208] Wright S (1943). Isolation-by-distance. Genetics.

[b209] Yakovlev IA, Fossdal CG, Johnsen O (2010). MicroRNAs, the epigenetic memory and climatic adaptation in Norway spruce. New Phytologist.

[b210] Yang LH, Rudolf VHW (2010). Phenology, ontogeny and the effects of climate change on the timing of species interactions. Ecology Letters.

[b211] Yeaman S, Jarvis A (2006). Regional heterogeneity and gene flow maintain variance in a quantitative trait within populations of lodgepole pine. Proceedings of the Royal Society B-Biological Sciences.

[b212] Yeaman S, Whitlock MC (2011). The genetic architecture of adaptation under migration-selection balance. Evolution.

[b213] Yu H, Luedeling E, Xu J (2010). Winter and spring warming result in delayed spring phenology on the Tibetan Plateau. Proceedings of the National Academy of Sciences of the United States of America.

