# Supplementary material for: Potential for evolutionary responses to climate change – evidence from tree populations
Source: Glob Chang Biol. 2013 Apr 3;19(6):1645–61. doi: 10.1111/gcb.12181 (PMC3664019; doi:10.1111/gcb.12181)
Supplement: Supplementary file 1 [file gcb0019-1645-SD1.doc]

***Supporting information references***

**Table 1**

Ballian D, Longauer R, Mikic T, Paule L, Kajba D, Gomory D (2006) Genetic structure of a rare European conifer, Serbian spruce (*Picea omorika* (Panc.) Purk.). *Plant Systematics and Evolution,* **260**, 53-63.

Belokon MM, Belokon YS, Politov DV, Altukhov YP (2005) Allozyme polymorphism of Swiss stone pine Pinus cembra L. in mountain populations of the Alps and the Eastern Carpathians. *Russian Journal of Genetics,* **41**, 1268-1280.

Boscherini G, Morgante M, Rossi P, Vendramin GG (1994) Allozyme and chloroplast DNA variation in Italian and Greek populations of Pinus leucodermis. *Heredity,* **73**, 284-290.

Ducci F, Proietti R, Favre JM (1999) Allozyme assessment of genetic diversity within the relic Sicilian fir *Abies nebrodensis* (Lojac.) Mattei. *Annals of Forest Science,* **56**, 345-355.

Fady B, Conkle MT (1993) Allozyme variation and possible phylogenetic implications in *Abies cephalonica* Loudon and some related Eastern Mediterranean firs. *Silvae Genetica,* **42**, 351-359.

Fallour D, Fady B, Lefevre F (1997) Study on isozyme variation in *Pinus pinea* L.: Evidence for low polymorphism. *Silvae Genetica,* **46**, 201-207.

Goncharenko GG, Padutov VE, Silin AE (1992) Population structure, gene diversity, and differentiaiton in natural populations of Cedar pines (Pinus subsetc. Cembrae, Pinacea) in the USSR. *Plant Systematics and Evolution,* **182**, 121-134.

Goncharenko GG, Silin AE, Padutov VE (1994) Allozyme variation in natural populations of Eurasian pines 3. Population structure, diversity, and gene flow in central and isolated populations of Pinus sylvestris L in Eastern Europe and Siberia. *Silvae Genetica,* **43**, 119-132.

Kara N, Korol L, Isik K, Schiller G (1997) Genetic diversity in *Pinus brutia* Ten.: Altitudinal variation. *Silvae Genetica,* **46**, 155-161.

Krutovskii KV, Bergmann F (1995) Introgressive hybridization and phylogenetic relationships between Norway, *Picea abies* (L) Karst., and Siberian, *Picea obovata* Lebeb., spruce species studied by isozyme loci. *Heredity,* **74**, 464-480.

Lewandowski A, Boratynski A, Mejnartowicz L (2000) Allozyme investigations on the genetic differentiation between closely related pines - Pinus sylvestris, P-mugo, P-uncinata, and P-uliginosa (Pinaceae). *Plant Systematics and Evolution,* **221**, 15-24.

Maier J (1992) Genetic variation in European larch (Larix decidua Mill.). *Annals of Forest Science,* **49**, 39-47.

Nikolic D, Tucic N (1983) Isoenzyme variation within and among populations of European black pine (Pinus nigra Arnold). *Silvae Genetica,* **32**, 80-89.

Salvador L, Alia R, Agundez D, Gil L (2000) Genetic variation and migration pathways of maritime pine (Pinus pinaster Ait) in the Iberian peninsula. *Theoretical and Applied Genetics,* **100**, 89-95.

Scaltsoyiannes A, Rohr R, Panetsos KP, Tsaktsira M (1994) Allozyme frequency distributions in 5 European populations of black pine (Pinus nigra Arnold) .1. Estimation of genetic variation within and among populations .2. Contribution of isozyme analysis to the taxonomic status of the species. *Silvae Genetica,* **43**, 20-30.

Scaltsoyiannes A, Tsaktsira M, Drouzas AD (1999) Allozyme differentiation in the Mediterranean firs (Abies, Pinaceae). A first comparative study with phylogenetic implications. *Plant Systematics and Evolution,* **216**, 289-307.

Scaltsoyiannes A, Tsaktsira M, Pasagiannis G, Tsoulpha P, Zhelev P, Iliev I, Rohr R (2009) Allozyme variation of European Black (Pinus nigra Arnold) and Scots pine (Pinus sylvestris L.) populations and implications on their evolution: A comparative study. *Journal of Biological Research-Thessaloniki,* **11**, 95-106.

Schiller G, Conkle MT, Grunwald C (1986) Local differentiation among Mediterranean populations of Aleppo pine in their isoenzymes. *Silvae Genetica,* **35**, 11-19.

Semerikov VL, Semerikov LF, Lascoux M (1999) Intra- and interspecific allozyme variability in Eurasian Larix Mill. species. *Heredity,* **82**, 193-204.

Semerikova SA, Semerikov VL (2006) Genetic variation and population differentiation in Siberian fir *Abies sibirica* Ledeb. inferred from allozyme markers. *Russian Journal of Genetics,* **42**, 636-644.

Slavov GT, Zhelev P (2004) Allozyme variation, differentiation, and inbreeding in populations of Pinus mugo in Bulgaria. *Canadian Journal of Forest Research-Revue Canadienne De Recherche Forestiere,* **34**, 2611-2617.

Tolun AA, Velioglu E, Cengel B, Kaya Z (2000) Genetic structure of black pine (*Pinus nigra* Arnold subspecies pallasiana) populations sampled from the Bolkar Mountains. *Silvae Genetica,* **49**, 113-119.

Zhelev P, Tzarska A (2009) Genetic diversity in the Bulgarian populations of *Pinus peuce* Grsb. In: *Conference on Breeding and Genetic Resources of Five-Needle Pines: Ecophysiology, Disease Resistance and Developmental Biology.* pp. 10-16, Yangyang, Republic of Koea, IUFRO.

**Table S1:** Description of the provenance trial studies.

Acevedo-Rodriguez R., Vargas-Hernandez J. J., Lopez-Upton J., Mendoza J. V. (2006) Effect of geographic origin and nutrition on shoot prenology of Mexican Douglas-Fir (Pseudotsuga sp.) seedlings. *Agrociencia,* **40**, 125-137.

Alberto F., Bouffier L., Louvet J. M., Lamy J. B., Delzon S., Kremer A. (2011) Adaptive responses for seed and leaf phenology in natural populations of sessile oak along an altitudinal gradient. *Journal of Evolutionary Biology,* **24**, 1442-1454.

Alia R., Gomez A., Agundez M. D., Bueno M. A., Notivol E. (2001a) Levels of genetic differentiation in Pinus halepensis Mill. in Spain using quantitative traits, isozymes, RAPDs and cp-microsatellites. In: *Genetic Response of Forest Systems to Changing Environmental Conditions.* (eds Mullerstarck G, Schubert R) pp Page.

Alia R., Moro-Serrano J., Notivol E. (2001b) Genetic variability of Scots pine (Pinus sylvestris) provenances in Spain: Growth traits and survival. *Silva Fennica,* **35**, 27-38.

Alonso J. G., Regneri S. M., Sanchez L. G. (2007) Absence of ecotypic differentiation in Mediterranean stone pine in a Spanish inland region. *Investigacion Agraria-Sistemas Y Recursos Forestales,* **16**, 253-261.

Andersson B., Fedorkov A. (2004) Longitudinal differences in Scots pine frost hardiness. *Silvae Genetica,* **53**, 76-80.

Aranda I., Alia R., Ortega U., Dantas A. K., Majada J. (2010) Intra-specific variability in biomass partitioning and carbon isotopic discrimination under moderate drought stress in seedlings from four Pinus pinaster populations. *Tree Genetics & Genomes,* **6**, 169-178.

Bariteau M. (1992) Geographic variation and stress adaptation of *Pinus halepensis* - *Pinus brutia* complex in Mediterranean conditions - Preliminary results of a provenance test in France. *Annales Des Sciences Forestieres,* **49**, 261-276.

Barnett P. E., Farmer R. E. (1980) Altitudinal variation in juvenile characteristics of southern Appalachian Black Cherry (*Prunus serotina* Ehrh.). *Silvae Genetica,* **29**, 157-160.

Benowicz A., El-Kassaby Y. A. (1999) Genetic variation in mountain hemlock (*Tsuga mertensiana* Bong.): quantitative and adaptive attributes. *Forest Ecology and Management,* **123**, 205-215.

Benowicz A., Guy R. D., El-Kassaby Y. A. (2000a) Geographic pattern of genetic variation in photosynthetic capacity and growth in two hardwood species from British Columbia. *Oecologia,* **123**, 168-174.

Benowicz A., El-Kassaby Y. A., Guy R. D., Ying C. C. (2000b) Sitka alder (*Alnus sinuata* Rydb.) genetic diversity in germination, frost hardiness and growth attributes. *Silvae Genetica,* **49**, 206-212.

Benowicz A., Guy R., Carlson M. R., El-Kassaby Y. A. (2001a) Genetic variation among paper birch (*Betula papyrifera* Marsh.) populations in germination, frost hardiness, gas exchange and growth. *Silvae Genetica,* **50**, 7-13.

Benowicz A., L'hirondelle S., El-Kassaby Y. A. (2001)b Patterns of genetic variation in mountain hemlock (Tsuga mertensiana (Bong.) Carr.) with respect to height growth and frost hardiness. *Forest Ecology and Management,* **154**, 23-33.

Beuker E. (1994) Adaptation to climatic changes of the timing of bud burst in populations of *Pinus sylvestris* L. and *Picea Abies* (L.) Karst. *Tree Physiology,* **14**, 961-970.

Beuker E., Valtonen E., Repo T. (1998) Seasonal variation in the frost hardiness of Scots pine and Norway spruce in old provenance experiments in Finland. *Forest Ecology and Management,* **107**, 87-98.

Bey C. F. (1979) Geographic variation in *Juglans nigra* in the midwestern United States. *Silvae Genetica,* **28**, 132-135.

Billington H. L., Pelham J. (1991) Genetic variation in the date of budburst in Scottish birch populations: Implications for climate change. *Functional Ecology,* **5**, 403-409.

Birot Y., Christophe C. (1983) Genetic structures and expected genetic gains from multitrait selection in wild populations of Douglas fir and Sitka spruce .1. Genetic variation between and within populations. *Silvae Genetica,* **32**, 141-151.

Blada I., Popescu F. (2007) Swiss stone pine provenance experiment in Romania: II. variation in growth and branching traits to age 14. *Silvae Genetica,* **56**, 148-158.

Bongarten B. C., Hanover J. W. (1986) Provenance variation in Blue spruce (*Picea pungens*) at 8 locations in the northern United States and Canada. *Silvae Genetica,* **35**, 67-74.

Bresson C. C., Vitasse Y., Kremer A., Delzon S. (2011) Acclimatization and adaptation in functional leaf traits of beech and oak trees along an altitudinal gradient. *Tree Physiology*, (*in press*).

Campbell R. K. (1979) Genecology of Douglas-Fir in a Watershed in the Oregon Cascades. *Ecology,* **60**, 1036-1050.

Campbell R. K., Ritland S. M. (1982) Regulation of seed germination timing by moist chilling in western Hemlock. *New Phytologist,* **92**, 173-182.

Cannell M. G. R., Willett S. C. (1976) Shoot growth phenology, dry-matter distribution and root-shoot ratios of provenances of *Populus trichocarpa*, *Picea sitchensis*, and *Pinus contorta* growing in Scotland. *Silvae Genetica,* **25**, 49-59.

Chmura D. J. (2006) Phenology differs among Norway spruce populations in relation to local variation in altitude of maternal stands in the Beskidy Mountains. *New Forests,* **32**, 21-31.

Chmura D. J., Rozkowski R. (2002) Variability of beech provenances in spring and autumn phenology. *Silvae Genetica,* **51**, 123-127.

Chuine I., Aitken S. N., Ying C. C. (2001) Temperature thresholds of shoot elongation in provenances of Pinus contorta. *Canadian Journal of Forest Research-Revue Canadienne De Recherche Forestiere,* **31**, 1444-1455.

Cordell S., Goldstein G., Mueller-Dombois D., Webb D., Vitousek P. M. (1998) Physiological and morphological variation in Metrosideros polymorpha, a dominant Hawaiian tree species, along an altitudinal gradient: the role of phenotypic plasticity. *Oecologia,* **113**, 188-196.

Court-Picon M., Gadbin-Henry C., Guibal F., Roux M. (2004) Dendrometry and morphometry of *Pinus pinea* L. in Lower Provence (France): adaptability and variability of provenances. *Forest Ecology and Management,* **194**, 319-333.

Daubree J. B., Kremer A. (1993) Genetic and phenological differentiation between introduced and natural populations of Quercus rubra L. *Ann. For. Sci.,* **50**, 271s-280s.

Davidson R. H., Edwards D. G. W., Sziklai O., Elkassaby Y. A. (1996) Genetic variation in germination parameters among populations of Pacific silver fir. *Silvae Genetica,* **45**, 165-171.

Deans J. D., Harvey F. J. (1995) Phenologies of 16 European provenances of sessile oak growing in Scotland. *Forestry,* **68**, 265-273.

Ducousso A., Louvet J. M., Jarret P., Kremer A. (2005) Geographic variations of sessile oaks in French provenance tests. *pp. 128-138 in Proceedings of the Joint Meeting of IUFRO working groups Genetic of oaks and Improvement and Silviculture of oaks, edited by R. Rogers, A. Ducousso and A. Kanazashi, FFPRI (Forestry and Forest Products Research Institute) Scientific Meeting Report 3, Tsukuba, Japan.*

Fady B. (1991a) Variation of vegetative flushing in *Abies cephalonica* Loudon in an experimental site. *Annales Des Sciences Forestieres,* **48**, 73-85.

Fady B. (1991b) Geographic and genetic variability of height growth in young *Abies cephalonica* Loud. *Annales Des Sciences Forestieres,* **48**, 279-295.

Farmer R. E. (1993) Latitudinal variation in height and phenology of balsam poplar. *Silvae Genetica,* **42**, 148-153.

Giertych M. (1979) Summary of results on European larch (*Larix decidua* Mill.) height growth in the IUFRO 1944 provenance experiment. *Silvae Genetica,* **28**, 244-256.

Gonzalez-Martinez S. C., Alia R., Gil L. (2002) Population genetic structure in a Mediterranean pine (Pinus pinaster Ait.): a comparison of allozyme markers and quantitative traits. *Heredity,* **89**, 199-206.

Gornall J. L., Guy R. D. (2007) Geographic variation in ecophysiological traits of black cottonwood (Populus trichocarpa). *Canadian Journal of Botany-Revue Canadienne De Botanique,* **85**, 1202-1213.

Green D. S. (2005) Adaptive strategies in seedlings of three co-occurring, ecologically distinct northern coniferous tree species across an elevational gradient. *Canadian Journal of Forest Research-Revue Canadienne De Recherche Forestiere,* **35**, 910-917.

Gulcu S., Ucler A. O. (2008) Genetic variation of Anatolian black pine (Pinus nigra Arnold. subsp pallasiana (Lamb.) holmboe) in the Lakes District of Turkey. *Silvae Genetica,* **57**, 1-5.

Hannerz M., Aitken S. N., King J. N., Budge S. (1999) Effects of genetic selection for growth on frost hardiness in western hemlock. *Canadian Journal of Forest Research-Revue Canadienne De Recherche Forestiere,* **29**, 509-516.

Hansen J. K., Larsen J. B. (2004) European silver fir (Abies alba Mill.) provenances from Calabria, southern Italy: 15-year results from Danish provenance field trials. *European Journal of Forest Research,* **123**, 127-138.

Hemery G. E., Savill P. S., Thakur A. (2005) Height growth and flushing in common walnut (Juglans regia L.): 5-year results from provenance trials in Great Britain. *Forestry,* **78**, 121-133.

Howe G. T., Hackett W. P., Furnier G. R., Klevorn R. E. (1995) Photoperiodic responses of a northern and southern ecotype of black cottonwood. *Physiologia Plantarum,* **93**, 695-708.

Hurme P., Repo T., Savolainen O., Paakkonen T. (1997) Climatic adaptation of bud set and frost hardiness in Scots pine (Pinus sylvestris). *Canadian Journal of Forest Research-Revue Canadienne De Recherche Forestiere,* **27**, 716-723.

Isik F., Keskin S., Mckeand S. E. (2000) Provenance variation and provenance-site interaction in Pinus brutia Ten.: Consequences of defining breeding zones. *Silvae Genetica,* **49**, 213-223.

Isik K., Kara N. (1997) Altitudinal variation in Pinus brutia Ten. and its implication in genetic conservation and seed transfers in southern Turkey. *Silvae Genetica,* **46**, 113-120.

Jaramillo-Correa J. P., Beaulieu J., Bousquet J. (2001) Contrasting evolutionary forces driving population structure at expressed sequence tag polymorphisms, allozymes and quantitative traits in white spruce. *Molecular Ecology,* **10**, 2729-2740.

Jensen J. S. (2000) Provenance variation in phenotypic traits in Quercus robur and Quercus petraea in Danish provenance trials. *Scandinavian Journal of Forest Research,* **15**, 297-308.

Jensen J. S., Deans J. D. (2004) Late autumn frost resistance of twelve north European provenances of Quercus species. *Scandinavian Journal of Forest Research,* **19**, 390-399.

Jensen J. S., Hansen J. K. (2008) Geographical variation in phenology of Quercus petraea (Matt.) Liebl and Quercus robur L. oak grown in a greenhouse. *Scandinavian Journal of Forest Research,* **23**, 179-188.

Kaya Z., Temerit A. (1994) Genetic structure of marginally located *Pinus nigra var. pallasiana* populations in Central Turkey. *Silvae Genetica,* **43**, 272-277.

Keller S. R., Soolanayakanahally R. Y., Guy R. D., Silim S. N., Olson M. S., Tiffin P. (2011) Climate-driven local adaptation of ecophysiology and phenology in balsam poplar, Populus balsamifera L. (Salicaceae). *American Journal of Botany,* **98**, 99-108.

Kremer A., Roussel G. (1986) Subdivision of shoot growth of maritime pine (*Pinus pinaster* Ait.) - Geographic variation of morphogenetical and phenological components. *Annales Des Sciences Forestieres,* **43**, 15-33.

Kriebel H. B., Bagley W. T., Deneke F. J., Funsch R. W., Roth P., Jokela J. J., Merritt C., Wright J. W. (1976) Geographic variation in Quercus rubra in north central United-states plantations. *Silvae Genetica,* **25**, 118-122.

Kuser J. E., Eberhardt J. C., Brennan E. G. (1989) Genecological variation in Pinus strobus - Growth-rate, date of fall needle-sheeding, and 2^nd^ winter needle retention. *Silvae Genetica,* **38**, 121-125.

Li P., Beaulieu J., Bousquet J. (1997a) Genetic structure and patterns of genetic variation among populations in eastern white spruce (Picea glauca). *Canadian Journal of Forest Research-Revue Canadienne De Recherche Forestiere,* **27**, 189-198.

Li P., Beaulieu J., Daoust G., Plourde A. (1997b) Patterns of adaptive genetic variation in eastern white pine (Pinus strobus) from Quebec. *Canadian Journal of Forest Research-Revue Canadienne De Recherche Forestiere,* **27**, 199-206.

Liepe K. (1993) Growth-chamber trial on frost hardiness and field trial on flushing of sessile oak (Quercus petraea Liebl). *Ann. For. Sci.,* **50**, 208s-214s.

Luo J. X., Zang R. G., Li C. Y. (2006) Physiological and morphological variations of Picea asperata populations originating from different altitudes in the mountains of southwestern China. *Forest Ecology and Management,* **221**, 285-290.

Luquez V., Hall D., Albrectsen B. R., Karlsson J., Ingvarsson P., Jansson S. (2008) Natural phenological variation in aspen (Populus tremula): the SwAsp collection. *Tree Genetics & Genomes,* **4**, 279-292.

Ma C. G. (1989) Geographic variation in *Pinus armandii* Franch. *Silvae Genetica,* **38**, 81-90.

Matyas C., Yeatman C. W. (1992) Effect of geographical transfer on groxth and survival off jack pine (*Pinus banksiana* Lamb) populations. *Silvae Genetica,* **41**, 370-376.

Mc Gee C. E. (1973) Is variation in budbreak of Red oak the result of heredity or environment? *Tree Improvement and Genetics Proceedings - Southern Forest Tree Improvement Conference - 1973*, 185-189.

Mimura M., Aitken S. N. (2007) Adaptive gradients and isolation-by-distance with postglacial migration in Picea sitchensis. *Heredity,* **99**, 224-232.

Morgenstern E. K. (1969) Genetic variation in seedlings of *Picea mariana* (Mill.) BSP. I. Correlation with ecological factors. *Silvae Genetica,* **18**, 151-167.

Myking T., Skroppa T. (2007) Variation in phenology and height increment of northern Ulmus glabra populations: Implications for conservation. *Scandinavian Journal of Forest Research,* **22**, 369-374.

Mylecraine K. A., Kuser J. E., Zimmermann G. L., Smouse P. E. (2005) Rangewide provenance variation in Atlantic white-cedar (Chamaecyparis thyoides): Early survival and growth in New Jersey and North Carolina plantations. *Forest Ecology and Management,* **216**, 91-104.

Nilsson J. E. (2001) Seasonal changes in phenological traits and cold hardiness of F1-populations from plus-trees of Pinus sylvestris and Pinus contorta of various geographical origins. *Scandinavian Journal of Forest Research,* **16**, 7-20.

Notivol E., Garcia-Gil M. R., Alia R., Savolainen O. (2007) Genetic variation of growth rhythm traits in the limits of a latitudinal cline in Scots pine. *Canadian Journal of Forest Research-Revue Canadienne De Recherche Forestiere,* **37**, 540-551.

Oleksyn J., Modrzynski J., Tjoelker M. G., Zytkowiak R., Reich P. B., Karolewski P. (1998) Growth and physiology of Picea abies populations from elevational transects: common garden evidence for altitudinal ecotypes and cold adaptation. *Functional Ecology,* **12**, 573-590.

Oleksyn J., Reich P. B., Zytkowiak R., Karolewski P., Tjoelker M. G. (2003) Nutrient conservation increases with latitude of origin in European Pinus sylvestris populations. *Oecologia,* **136**, 220-235.

Portefaix C. (1989) Exploration of genetic variability of 5 natural stands of Corsican pine (*Pinus nigra* ssp. *laricio* var. *corsicana* Loud.). *Annales Des Sciences Forestieres,* **46**, 217-232.

Premoli A. C., Brewer C. A. (2007) Environmental v. genetically driven variation in ecophysiological traits of Nothofagus pumilio from contrasting elevations. *Australian Journal of Botany,* **55**, 585-591.

Premoli A. C., Raffaele E., Mathiasen P. (2007) Morphological and phenological differences in Nothofagus pumilio from contrasting elevations: Evidence from a common garden. *Austral Ecology,* **32**, 515-523.

Rehfeldt G. E. (1978) Genetic differentiaiton of Douglas fir populations from the northern Rocky Mountains. *Ecology,* **59**, 1264-1270.

Rehfeldt G. E. (1979) Ecological adaptations in Douglas fir (Pseudotsuga menziesii var glauca) populations .1. North Idao and northeast Washington. *Heredity,* **43**, 383-397.

Rehfeldt G. E. (1980) Cold acclimatation in populations of Pinus contorta from the northern Rocky Mountains. *Botanical Gazette,* **141**, 458-463.

Rehfeldt G. E. (1982) Differentiation of Larix occidentalis populations from the northern Rocky Mountains. *Silvae Genetica,* **31**, 13-19.

Rehfeldt G. E. (1988) Ecological genetics of Pinus contorta from the Rocky Mountains (USA) - A synthesis. *Silvae Genetica,* **37**, 131-135.

Rehfeldt G. E. (1993) Genetic variation in the Ponderosae of the southwest. *American Journal of Botany,* **80**, 330-343.

Rehfeldt G. E. (1995) Genetic variation, climate models and the ecological genetics of Larix occidentalis. *Forest Ecology and Management,* **78**, 21-37.

Rehfeldt G. E., Hoff R. J., Steinhoff R. J. (1984) Geographic patterns of genetic variation in Pinus monticola. *Botanical Gazette,* **145**, 229-239.

Rehfeldt G. E., Wykoff W. R. (1981) Periodicity in shoot elongation among populations of Pinus contorta from the northern Rocky Mountains. *Annals of Botany,* **48**, 371-377.

Reich P. B., Oleksyn J., Tjoelker M. G. (1996) Needle respiration and nitrogen concentration in Scots Pine populations from a broad latitudinal range: A common garden test with field-grown trees. *Functional Ecology,* **10**, 768-776.

Rweyongeza D. M., Yang R. C., Dhir N. K., Barnhardt L. K., Hansen C. (2007) Genetic variation and climatic impacts on survival and growth of white spruce in Alberta, Canada. *Silvae Genetica,* **56**, 117-127.

Saenz-Romero C., Guzman-Reyna R. R., Rehfeldt G. E. (2006) Altitudinal genetic variation among Pinus oocarpa populations in Michoacan, Mexico - Implications for seed zoning, conservation, tree breeding and global warming. *Forest Ecology and Management,* **229**, 340-350.

Sanchez-Gomez D., Majada J., Alia R., Feito I., Aranda I. (2010) Intraspecific variation in growth and allocation patterns in seedlings of Pinus pinaster Ait. submitted to contrasting watering regimes: can water availability explain regional variation? *Annals of Forest Science,* **67**.

Savolainen O., Bokma F., Garcia-Gil R., Komulainen P., Repo T. (2004) Genetic variation in cessation of growth and frost hardiness and consequences for adaptation of Pinus sylvestris to climatic changes. *Forest Ecology and Management,* **197**, 79-89.

Simpson D. G. (1994) Seasonal and geographical origin effects on cold hardiness of White spruce buds, foliage, and stems. *Canadian Journal of Forest Research-Revue Canadienne De Recherche Forestiere,* **24**, 1066-1070.

Skroppa T., Magnussen S. (1993) Provenance variation in shoot growth components of Norway spruce. *Silvae Genetica,* **42**, 111-120.

Sogaard G., Johnsen O., Nilsen J., Junttila O. (2008) Climatic control of bud burst in young seedlings of nine provenances of Norway spruce. *Tree Physiology,* **28**, 311-320.

Soolanayakanahally R. Y., Guy R. D., Silim S. N., Drewes E. C., Schroeder W. R. (2009) Enhanced assimilation rate and water use efficiency with latitude through increased photosynthetic capacity and internal conductance in balsam poplar (Populus balsamifera L.). *Plant Cell and Environment,* **32**, 1821-1832.

Sorensen F. C. (1983) Geographic variation in seedling Douglas fir (*Pseudotsuga menziesii*) from the western Siskiyou Mountains of Oregon. *Ecology,* **64**, 696-702.

St Clair J. B., Mandel N. L., Vance-Boland K. W. (2005) Genecology of Douglas fir in western Oregon and Washington. *Annals of Botany,* **96**, 1199-1214.

Steiner K. C. (1979a) Patterns of variation in bud-burst timing among populations in several *Pinus* species. *Silvae Genetica,* **28**, 185-194.

Steiner K. C. (1979b) Variation in bud-burst timing among populations of interior Douglas fir. *Silvae Genetica,* **28**, 76-79.

Sundblad L. G., Andersson B. (1995) No difference in frost hardiness between high and low altitude *Pinus sylvestris* (L.) offspring. *Scandinavian Journal of Forest Research,* **10**, 22-26.

Townsend A. M., Wright J. W., Kwolek W. F., Beineke W. F., Lester D. T., Mohn C. A., Dodge A. F. (1979) Geographic variation in young Red maple grown in north central United States. *Silvae Genetica,* **28**, 33-36.

Treesnips final report (2006) Developing single nucleotide polymorphism (SNP) markers for adaptive variation in forest trees Final report on the 5^th^ framework project. Available at: https://wiki.oulu.fi/download/attachments/13382380/Treesnips-final.pdf?version=1&modificationDate=1352295252271

Vihera-Aarnio A., Hakkinen R., Partanen J., Luomajoki A., Koski V. (2005) Effects of seed origin and sowing time on timing of height growth cessation of Betula pendula seedlings. *Tree Physiology,* **25**, 101-108.

Vitasse Y., Delzon S., Bresson C. C., Michalet R., Kremer A. (2009) Altitudinal differentiation in growth and phenology among populations of temperate-zone tree species growing in a common garden. *Canadian Journal of Forest Research-Revue Canadienne De Recherche Forestiere,* **39**, 1259-1269.

Viveros-Viveros H., Saenz-Romero C., Lopez-Upton J., Vargas-Hernandez J. J. (2005) Altitudinal genetic variation in plant growth of Pinus pseudostrobus Lindl. in field testing. *Agrociencia,* **39**, 575-587.

Voltas J., Chambel M., Prada M., Ferrio J. (2008) Climate-related variability in carbon and oxygen stable isotopes among populations of Aleppo pine grown in common-garden tests. *Trees,* **22**, 759-769.

Vonwuehlisch G., Krusche D., Muhs H. J. (1995) Variation in temperature sum requirement for flushing of beech provenances. *Silvae Genetica,* **44**, 343-346.

Wilcox M. D., Ledgard N. J. (1983) Provenance variation in the New Zealand species of Nothofagus. *New Zealand Journal of Ecology,* **6**, 19-31.

Worrall J. (1983) Temperature - bud-burst relationships in Amabilis and Subalpine fir provenance tests replicated at different elevations. *Silvae Genetica,* **32**, 203-209.

Worrell R., Cundall E. P., Malcolm D. C., Ennos R. A. (2000) Variation among seed sources of silver birch in Scotland. *Forestry,* **73**, 419-435.

Zhang J. W., Marshall J. D. (1995) Variation in carbon-isotope discrimination and photosynthetic gas-exchange among populations of Pseudotsuga menziesii and Pinus ponderosa in different environments. *Functional Ecology,* **9**, 402-412.

Zhang J. W., Marshall J. D., Jaquish B. C. (1993) Genetic differentiation in carbon isotope discrimination and gas-exchange in Pseudotsuga menziesii - a common garden experiment. *Oecologia,* **93**, 80-87.

**Table S2:** Nucleotide diversity estimates per gene.

Breen A. L., Glenn E., Yeager A., Olson M. S. (2009) Nucleotide diversity among natural populations of a North American poplar (Populus balsamifera, Salicaceae). *New Phytologist,* **182**, 763-773.

Brown G. R., Gill G. P., Kuntz R. J., Langley C. H., Neale D. B. (2004) Nucleotide diversity and linkage disequilibrium in loblolly pine. *Proceedings of the National Academy of Sciences of the United States of America,* **101**, 15255-15260.

Chen J., Kallman T., Gyllenstrand N., Lascoux M. (2010) New insights on the speciation history and nucleotide diversity of three boreal spruce species and a Tertiary relict. *Heredity,* **104**, 3-14.

Chu Y. G., Su X. H., Huang Q. J., Zhang X. H. (2009) Patterns of DNA sequence variation at candidate gene loci in black poplar (Populus nigra L.) as revealed by single nucleotide polymorphisms. *Genetica,* **137**, 141-150.

Derory J., Scotti-Saintagne C., Bertocchi E., Le Dantec L., Graignic N., Jauffres A., Casasoli M., Chancerel E. (2010) Contrasting relationships between the diversity of candidate genes and variation of bud burst in natural and segregating populations of European oaks. *Heredity,* **104**, 438-448.

Eckert A. J., Wegrzyn J. L., Pande B., Jermstad K. D., Lee J. M., Liechty J. D., Tearse B. R., Krutovsky K. V. (2009) Multilocus Patterns of Nucleotide Diversity and Divergence Reveal Positive Selection at Candidate Genes Related to Cold Hardiness in Coastal Douglas Fir (Pseudotsuga menziesii var. menziesii). *Genetics,* **183**, 289-298.

Eveno E., Collada C., Guevara M. A., Leger V., Soto A., Diaz L., Leger P., Gonzalez-Martinez S. C. (2008) Contrasting patterns of selection at Pinus pinaster Ait. drought stress candidate genes as revealed by genetic differentiation analyses. *Molecular Biology and Evolution,* **25**, 417-437.

Fujimoto A., Kado T., Yoshimaru H., Tsumura Y., Tachida H. (2008) Adaptive and slightly deleterious evolution in a conifer, Cryptomeria japonica. *Journal of Molecular Evolution,* **67**, 201-210.

Gilchrist E. J., Haughn G. W., Ying C. C., Otto S. P., Zhuang J., Cheung D., Hamberger B., Aboutorabi F. (2006) Use of Ecotilling as an efficient SNP discovery tool to survey genetic variation in wild populations of Populus trichocarpa. *Molecular Ecology,* **15**, 1367-1378.

Gonzalez-Martinez S. C., Ersoz E., Brown G. R., Wheeler N. C., Neale D. B. (2006) DNA sequence variation and selection of tag single-nucleotide polymorphisms at candidate genes for drought-stress response in Pinus taeda L. *Genetics,* **172**, 1915-1926.

Heuertz M., De Paoli E., Kallman T., Larsson H., Jurman I., Morgante M., Lascoux M., Gyllenstrand N. (2006) Multilocus patterns of nucleotide diversity, linkage disequilibrium and demographic history of Norway spruce [Picea abies (L.) Karst]. *Genetics,* **174**, 2095-2105.

Ingvarsson P. K. (2005a) Nucleotide polymorphism and linkage disequilbrium within and among natural populations of European Aspen (Populus tremula L., Salicaceae). *Genetics,* **169**, 945-953.

Ingvarsson P. K. (2005b) Molecular population genetics of herbivore-induced protease inhibitor genes in European Aspen (Populus tremula L., Salicaceae). *Molecular Biology and Evolution,* **22**, 1802-1812.

Ingvarsson P. K., Garcia M. V., Hall D., Luquez V., Jansson S. (2006) Clinal variation in phyB2, a candidate gene for day-length-induced growth cessation and bud set, across a latitudinal gradient in European aspen (Populus tremula). *Genetics,* **172**, 1845-1853.

Jarvinen P., Lemmetyinen J., Savolainen O., Sopanen T. (2003) DNA sequence variation in BpMADS2 gene in two populations of Betula pendula. *Molecular Ecology,* **12**, 369-384.

Kado T., Matsumoto A., Ujino-Ihara T., Tsumura Y. (2008) Amounts and patterns of nucleotide variation within and between two Japanese conifers, sugi (Cryptomeria japonica) and hinoki (Chamaecyparis obtusa) (Cupressaceae sensu lato). *Tree Genetics & Genomes,* **4**, 133-141.

Kado T., Yoshimaru H., Tsumura Y., Tachida H. (2003) DNA Variation in a Conifer, Cryptomeria japonica (Cupressaceae sensu lato). *Genetics,* **164**, 1547-1559.

Krutovsky K. V., Neale D. B. (2005) Nucleotide diversity and linkage disequilibrium in cold-hardiness- and wood quality-related candidate genes in Douglas fir. *Genetics,* **171**, 2029-2041.

Ma X. F., Szmidt A. E., Wang X. R. (2006) Genetic structure and evolutionary history of a diploid hybrid pine Pinus densata inferred from the nucleotide variation at seven gene loci. *Molecular Biology and Evolution,* **23**, 807-816.

Marroni F., Pinosio S., Zaina G., Fogolari F., Felice N., Cattonaro F., Morgante M. (2011) Nucleotide diversity and linkage disequilibrium in Populus nigra cinnamyl alcohol dehydrogenase (CAD4) gene. *Tree Genetics & Genomes,* **7**, 1011-1023.

Namroud M. C., Guillet-Claude C., Mackay J., Isabel N., Bousquet J. (2010) Molecular Evolution of Regulatory Genes in Spruces from Different Species and Continents: Heterogeneous Patterns of Linkage Disequilibrium and Selection but Correlated Recent Demographic Changes. *Journal of Molecular Evolution,* **70**, 371-386.

Olson M. S., Robertson A. L., Takebayashi N., Silim S., Schroeder W. R., Tiffin P. (2010) Nucleotide diversity and linkage disequilibrium in balsam poplar (Populus balsamifera). *New Phytologist,* **186**, 526-536.

Palme A. E., Wright M., Savolainen O. (2008) Patterns of Divergence among Conifer ESTs and Polymorphism in Pinus sylvestris Identify Putative Selective Sweeps. *Molecular Biology and Evolution,* **25**, 2567-2577.

Pot D., Mcmillan L., Echt C., Le Provost G., Garnier-Gere P., Cato S., Plomion C. (2005) Nucleotide variation in genes involved in wood formation in two pine species. *New Phytologist,* **167**, 101-112.

Quang N. D., Ikeda S., Harada K. (2008) Nucleotide variation in Quercus crispula Blume. *Heredity,* **101**, 166-174.

Wachowiak W., Balk P. A., Savolainen O. (2009) Search for nucleotide diversity patterns of local adaptation in dehydrins and other cold-related candidate genes in Scots pine (Pinus sylvestris L.). *Tree Genetics & Genomes,* **5**, 117-132.

Wachowiak W., Salmela M. J., Ennos R. A., Iason G., Cavers S. (2011) High genetic diversity at the extreme range edge: nucleotide variation at nuclear loci in Scots pine (Pinus sylvestris L.) in Scotland. *Heredity,* **106**, 775-787.

**Table S3:** SNP effect sizes in association studies.

Beaulieu J., Doerksen T., Boyle B., Clement S., Deslauriers M., Beauseigle S., Blais S., Poulin P. L. (2011) Association Genetics of Wood Physical Traits in the Conifer White Spruce and Relationships With Gene Expression. *Genetics,* **188**, 197-U329.

Cumbie W. P., Eckert A., Wegrzyn J., Whetten R., Neale D., Goldfarb B. (2011) Association genetics of carbon isotope discrimination, height and foliar nitrogen in a natural population of Pinus taeda L. *Heredity,* **107**, 105-114.

Dillon S. K., Nolan M., Li W., Bell C., Wu H. X., Southerton S. G. (2010) Allelic Variation in Cell Wall Candidate Genes Affecting Solid Wood Properties in Natural Populations and Land Races of Pinus radiata. *Genetics,* **185**, 1477-U1545.

Eckert A. J., Bower A. D., Wegrzyn J. L., Pande B., Jermstad K. D., Krutovsky K. V., Clair J. B. S., Neale D. B. (2009) Association Genetics of Coastal Douglas Fir (Pseudotsuga menziesu var. menziesii, Pinaceae). I. Cold-Hardiness Related Traits. *Genetics,* **182**, 1289-1302.

Gonzalez-Martinez S. C., Wheeler N. C., Ersoz E., Nelson C. D., Neale D. B. (2007) Association genetics in Pinus taeda L. I. Wood property traits. *Genetics,* **175**, 399-409.

Holliday J. A., Ritland K., Aitken S. N. (2010) Widespread, ecologically relevant genetic markers developed from association mapping of climate-related traits in Sitka spruce (*Picea sitchensis*). *New Phytol,* **188**, 501-514.

Ingvarsson P. K., Garcia M. V., Luquez V., Hall D., Jansson S. (2008) Nucleotide polymoirphism and phenotypic associations within and around the phytochrome B2 locus in European aspen (Populus tremula, Salicaceae). *Genetics,* **178**, 2217-2226.

Ma X. F., Hall D., St Onge K. R., Jansson S., Ingvarsson P. K. (2010) Genetic Differentiation, Clinal Variation and Phenotypic Associations With Growth Cessation Across the Populus tremula Photoperiodic Pathway. *Genetics,* **186**, 1033-1044.

Quesada T., Gopal V., Cumbie W. P., Eckert A. J., Wegrzyn J. L., Neale D. B., Goldfarb B., Huber D. A. (2010) Association Mapping of Quantitative Disease Resistance in a Natural Population of Loblolly Pine (Pinus taeda L.). *Genetics,* **186**, 677-U336.

Thumma B. R., Nolan M. R., Evans R., Moran G. F. (2005) Polymorphisms in cinnamoyl CoA reductase (CCR) are associated with variation in microfibril angle in Eucalyptus spp. *Genetics,* **171**, 1257-1265.

Wegrzyn J. L., Eckert A. J., Choi M., Lee J. M., Stanton B. J., Sykes R., Davis M. F., Tsai C. J. (2010) Association genetics of traits controlling lignin and cellulose biosynthesis in black cottonwood (Populus trichocarpa, Salicaceae) secondary xylem. *New Phytologist,* **188**, 515-532.
